# Inferring excitation-inhibition dynamics using a maximum entropy model unifying brain structure and function

**DOI:** 10.1162/netn_a_00220

**Published:** 2022-06-01

**Authors:** Igor Fortel, Mitchell Butler, Laura E. Korthauer, Liang Zhan, Olusola Ajilore, Anastasios Sidiropoulos, Yichao Wu, Ira Driscoll, Dan Schonfeld, Alex Leow

**Affiliations:** Department of Bioengineering, University of Illinois at Chicago, Chicago, IL, USA; Department of Psychology, University of Wisconsin–Milwaukee, Milwaukee, WI, USA; Warren Alpert Medical School, Brown University, Providence, RI, USA; Department of Electrical and Computer Engineering, University of Pittsburgh, Pittsburgh, PA, USA; Department of Psychiatry, University of Illinois at Chicago, Chicago, IL, USA; Department of Computer Science, University of Illinois at Chicago, Chicago, IL, USA; Department of Math, Statistics, and Computer Science, University of Illinois at Chicago, Chicago, IL, USA; Department of Electrical and Computer Engineering, University of Illinois at Chicago, Chicago, IL, USA

**Keywords:** Functional connectivity, Structural connectome, Ising model, Brain criticality, Excitation-inhibition balance, Maximum entropy

## Abstract

Neural activity coordinated across different scales from neuronal circuits to large-scale brain networks gives rise to complex cognitive functions. Bridging the gap between micro- and macroscale processes, we present a novel framework based on the maximum entropy model to infer a hybrid resting-state structural connectome, representing functional interactions constrained by structural connectivity. We demonstrate that the structurally informed network outperforms the unconstrained model in simulating brain dynamics, wherein by constraining the inference model with the network structure we may improve the estimation of pairwise BOLD signal interactions. Further, we simulate brain network dynamics using Monte Carlo simulations with the new hybrid connectome to probe connectome-level differences in excitation-inhibition balance between apolipoprotein E (APOE)-ε4 carriers and noncarriers. Our results reveal sex differences among APOE-ε4 carriers in functional dynamics at criticality; specifically, female carriers appear to exhibit a lower tolerance to network disruptions resulting from increased excitatory interactions. In sum, the new multimodal network explored here enables analysis of brain dynamics through the integration of structure and function, providing insight into the complex interactions underlying neural activity such as the balance of excitation and inhibition.

## INTRODUCTION

The brain is a complex dynamical system whose functional properties are largely determined by the characteristics of its neurons and patterns of synaptic connectivity, resulting in a balance of excitatory (E) and inhibitory (I) interactions. For example, if the number of neurons that are coactivated from one signal is too high (increased excitation), the result is wide-scale activations and errant signal propagation across the brain’s subnetworks. On the other hand, if the number of coactivated neurons is too low (increased inhibition), the propagation of the signal may diminish too quickly, limiting information transfer. The dynamical balance between excitation and inhibition is important for adjusting neural input/output relationships in cortical networks and regulating the dynamic range of their responses to stimuli (Kinouchi & Copelli, 2006) as well as the optimal dynamic range where information capacity and transfer are maximized (Shew et al., 2011). This is the central thesis of the criticality hypothesis, a phenomenon that suggests that neural networks and many aspects of brain activity self-organize into a unique configuration, sometimes called a critical state (Wilting & Priesemann, 2019). This state represents the transition of complex dynamical systems like the brain from order (balanced excitation-inhibition) to disorder (disrupted excitation-inhibition balance) and has found applications in many scientific domains, including neuroscience and clinical neurology (Cocchi et al., 2017; Hahn et al., 2017; Sornette, 2006; Tagliazucchi, 2017). Studies have demonstrated that the cortex operates near criticality during neuronal signaling (Beggs & Plenz, 2003; Hahn et al., 2017; Shew et al., 2009), as well as in studies utilizing blood oxygen level–dependent (BOLD) signals extracted from fMRI imaging (Haimovici et al., 2013; Lombardi et al., 2017; Rabuffo et al., 2021; Tagliazucchi et al., 2012). In fact, there is growing evidence from animal models and whole-cell recordings supporting the hypothesis that synaptic dysfunction leading to neuronal hyperexcitation may represent some of the earliest changes in the progression of neurodegenerative disease like Alzheimer’s disease (AD; Busche & Konnerth, 2016; Palop et al., 2007; Petrache et al., 2019; Ren et al., 2018). However, the major challenge with early detection and intervention is that both normal aging and AD are associated with alterations to neural structure and function (McDonald et al., 2009; Schuff et al., 1999). This includes regional hypometabolism (Chételat et al., 2013; Curiati et al., 2011), white matter (WM) changes (Barrick et al., 2010; Michielse et al., 2010), Aβ deposition (Rodrigue et al., 2012; Rowe et al., 2010), and disrupted resting-state functional connectivity (Damoiseaux et al., 2008; Sheline et al., 2010; Wang et al., 2006). To improve our understanding of neurodegenerative diseases (accounting for major factors such as age, sex, or genetic phenotypes) and improve early detection, we investigate a model that can integrate microscale principles at a connectome level to bridge the gap between cell- to network-level degeneration. However, we acknowledge that some abstraction is required in this strategy; in models of large-scale effects, physiological information may be more abstract, and details of cellular processes potentially lost. While this may seem counterintuitive from a biological perspective, it is necessary for describing higher-level phenomena informed by MRI neuroimaging.

To this end, in this paper we introduce a method based on statistical physics to jointly model both brain structure and function via a pairwise maximum entropy model (pMEM). Our framework is inspired by the Ising model representation of brain dynamics whereby self-organized patterns of connectivity are formed through the spontaneous fluctuations of random spins (Reichl & Luscombe, 1999). This model has been used to characterize complex microscale dynamics of the human brain (Deco et al., 2008; Kadirvelu et al., 2017; Ostojic & Brunel, 2011; Tkačik et al., 2015), as well as macroscale interactions (Ezaki et al., 2017; Marinazzo et al., 2014; Nghiem et al., 2018; Niu et al., 2019; Nuzzi et al., 2020; Schneidman et al., 2006). Unconstrained maximum entropy models (MEM) have been shown to accurately represent spatiotemporal coactivations in neuronal spike trains (Roudi et al., 2009; Schneidman et al., 2006; Shlens et al., 2006) as well as patterns of BOLD activity (Ashourvan et al., 2017; Cocco et al., 2017; Ezaki et al., 2020; Watanabe et al., 2013). In fact, Zanoci et al. (2019) recently showed that the Ising model captures collective neuronal behavior during wakefulness, light sleep, and deep sleep when both excitatory (E) and inhibitory (I) neurons are modeled. Further, at the macroscale, Ashourvan et al. (2021) recently developed a maximum entropy–based framework that derives functional connectivity measures from intracranial EEG recordings; their findings suggest that structural connections in the brain give rise to large-scale patterns of functional connectivity by promoting coactivation between connected structures. Thus, MEM may be an ideal tool to model functional connectivity and ultimately link microscale interactions (such as excitation and inhibition in neuronal circuits) to the functional connectome (FC) captured through fMRI BOLD activity.

Described as a function-by-structure embedding (FSE), our model infers the organization of functional connectivity from global activity patterns (i.e., simultaneously considering the activity of more than two brain regions) constrained to the structural connectome. We present a robust numerical approach for our model, optimizing a constrained maximum likelihood estimation. The use of a structural connectome to inform the modeling of BOLD activity is motivated by a strong link between fMRI-based functional connectivity and white matter–based structural connectivity (Bettinardi et al., 2017; Honey et al., 2009; Shen et al., 2015). These studies suggest that models of functional dynamics should also be governed by the underlying structure to include direct and indirect connections between brain regions. Thus, if our model accurately describes large-scale brain activity patterns during rest, it will provide a much richer representation of functional interactions governing global dynamics that may give rise to hyperexcitation. With our framework we construct hybrid resting-state structural connectomes (rs-SC) for a group of 76 middle-aged and cognitively intact individuals. These unique structural networks are informed by a spin-glass-like Ising model, whose dynamics resemble that of traditional FC. We demonstrate that our new structurally informed networks can consistently and accurately reconstruct observed BOLD correlations. Investigating macroscale brain dynamics through the lens of statistical physics allows us to infer computationally the nature of resting-state activity (corresponding to inhibition or excitation) and probe potential disruptions to E/I balance that may lead to hyperexcitation and subsequent increased vulnerability to neurodegeneration. To evaluate this phenomenon, we create subgroups of 38 age- and sex-matched individuals based on whether one is a carrier of the apolipoprotein E (APOE)-ε4 allele, a well-known genetic risk factor of AD. Recent studies have shown that APOE-ε4 may contribute directly to early neuronal dysfunction, either directly via modification of the excitation/inhibition balance or linked with amyloid deposition (Bi et al., 2020; Koelewijn et al., 2019; Nuriel et al., 2017; Stargardt et al., 2015). Using our new hybrid rs-SC, we investigate the relationship between E/I balance and criticality in these two groups. We hypothesized that because of a shift in E/I balance towards hyperexcitation, the female APOE-ε4 carrier group would exhibit a lower tolerance to perturbations in the network when simulating brain dynamics using Monte Carlo simulations of the Ising model as compared with the female noncarrier group. Herein we aim to demonstrate that an increase in excitatory interactions at the connectome level, identified using our new hybrid connectome, may provide new evidence of vulnerability among females to AD neuropathology due to disruptions in E/I balance.

## RESULTS

### Constructing a Function-by-Structure Embedding Using a Constrained Maximum Likelihood Estimation

In constructing the function-by-structure embedding (FSE), we begin with the unconstrained pairwise maximum entropy model (pMEM) as described in the Methods section. The pMEM is sometimes referred to as the inverse Ising model, where the pairwise interactions (represented as *J*_*i*,*j*_, with *i* and *j* representing regions of interest, ROIs, in the brain network) are inferred from the observed data (BOLD time series). As the model assumes binary data, we binarized the resting-state fMRI signals obtained from the 76 cognitively intact middle-aged subjects. The binarized activity pattern of *N* = 80 ROIs at time *t* (*t* = 1, 2, …, *t*_*max*_; *t*_*max*_ = 236) is denoted ***s***(*t*) = *s*_1_(*t*), *s*_2_(*t*), …, *s*_*N*_(*t*) ∈ {−1, +1}^*N*^. Note that *t*_*max*_ is determined as a result of the 8-min fMRI scan time with TR = 2 s (see the Methods section). Here *s*_1_(*t*) = ± 1 indicates that an ROI is either active (+1) or inactive (−1). First, the time series goes through a z-score normalization procedure, resulting in zero mean and unitary variance. To assess the sensitivity of our results to thresholding, we tested thresholds of 0 and ±1 *SD*. The results of this assessment will be presented in the section called Determining Parameters for Generating the Optimal Resting-State Structural Connectome. For the unconstrained pMEM, we fit the following probability distribution to all 76 subjects by maximizing a pseudolikelihood (see the Methods section): *Pr*(***s***) = exp (−*βH*(*s*))/*Z*, where *H*(***s***) = −∑_<*i* *j*>_ *J*_*i*,*j*_*s*_*i*_*s*_*j*_, with *i*, *j* ∈ [1, 2, …, *k*] is the Hamiltonian function describing the energy of the system, and *Z* = ∑_***s***_ exp(−*βH*(***s***)) is the partition function. Here, the spin configuration ***s*** is defined as the column vector **s** = [*s*_1_, *s*_2_, …, *s*_*N*_]^*t*_*max*_^, where *s*_*i*_ and *s*_*j*_ are the spin states of region *i* and *j*, and *J*_*i*,*j*_ represents a pairwise interaction between those regions. Traditionally, the Hamiltonian includes a term for external influences that we assume to be zero for resting-state data. We use the unconstrained pMEM as a control for comparison purposes. In our approach, we hypothesized that the interaction *J*_*i*,*j*_ between two regions should be directly linked back to the diffusion MRI-derived structural connectivity between them as informed by tractography, so we add a constraint to the Hamiltonian function as follows:

$$H\left(s\right)=-\sum_{i<j}J_{i,j}s_{i}s_{j},\;\text{such}\;\text{that}\;\left|J_{i,j}\right|\propto W_{i,j},$$ (1)

where *W*_*i*,*j*_ is the structural connectivity between pairs of ROIs. This ensures that in the pseudolikelihood estimation of ***J***, we constrain it with the structural connectivity (under the assumption that structural connectivity informs spin models governing brain dynamics). Thus, the optimal interaction matrix ***J*** is derived by maximizing the pseudolikelihood function as follows (Besag, 1975, 1977):

$${𝓛}_{\text{pseudo}}\left(J,\beta \right)=\prod_{t=1}^{t_{\max}}\prod_{i=1}^{k}\text{Pr}\left(s_{i}\left(t\right)|J,\beta ,s_{-i\left(t\right)}\right).$$ (2)

Pseudolikelihood substitutes *Pr*(***s***) by the product of the conditional probabilities $\tilde{p}$ = *Pr*(*s*_*i*_(*t*)|***J***, *β*, ***s***_−*i*(*t*)_), observing one element *s*_*i*_(*t*) with all the other elements (denoted ***s***_−*i*(*t*)_) fixed. To ensure that the magnitude of the coupling interactions is scaled relative to structural connectivity, the constraint is formulated as |*J*_*i*,*j*_| ∼ = *μW*_*i*,*j*_, where *μ* is a normalization constant and *W*_*i*,*j*_ is the structural connectivity between ROI pairs. Without loss of generality, we assume that *μ* = 1 with appropriate normalization. We therefore present a penalty-based optimization scheme to maximize the constrained log-pseudolikelihood function as follows:

$$\ell \left(J,\beta \right)=\frac{1}{t_{\max}}\text{ln}\;{𝓛}_{\text{pseudo}}\left(J,\beta \right)-\frac{\lambda }{2}\sum_{i<j}{\left(J_{i,j}-\text{sgn}\left(J_{i,j}\right)W_{i,j}\right)}^{2}.$$ (3)

The pseudolikelihood component $\frac{1}{t_{\max}}$ ln 𝓛_*pseudo*_(***J***, *β*) expands to the following:

$$\frac{1}{t_{\max}}\;\sum_{t=1}^{t_{\max}}\sum_{i=1}^{N}\ln\left(\frac{\exp\left(\beta \sum_{k=1}^{N}J_{i,k}s_{i}\left(t\right)s_{k}\left(t\right)\right)}{\exp\left(\beta \sum_{k=1}^{N}J_{i,k}s_{k}\left(t\right)\right)+\exp\left(-\beta \sum_{k=1}^{N}J_{i,k}s_{k}\left(t\right)\right)}\right).$$ (4)

We formulate the probability distribution based on the Boltzmann distribution under pseudolikelihood conditions. Thus, the numerator describes the energy of the system, while the denominator is the sum of all possible energies. Hence, there are only two terms in the denominator since *s*_*i*_(*t*) is binary (one positive, and one negative). The likelihood function may be simplified by setting *C*_*i*_(*t*) = β $\sum_{m=1}^{k}$ *J*_*i*,*m*_*s*_*m*_(*t*), resulting in the following formulation:

$$\ell \left(J,\beta \right)=\frac{1}{t_{\max}}\;\sum_{t=1}^{t_{\max}}\sum_{i=1}^{N}C_{i}\left(t\right)s_{i}\left(t\right)-\ln\left(\text{exp}\left(C_{i}\left(t\right)\right)+\text{exp}\left(-C_{i}\left(t\right)\right)\right)-\frac{\lambda }{2}\sum_{i<j}{\left(J_{i,j}-\text{sgn}\left(J_{i,j}\right)W_{i,j}\right)}^{2}.$$ (5)

**Figure 1.**
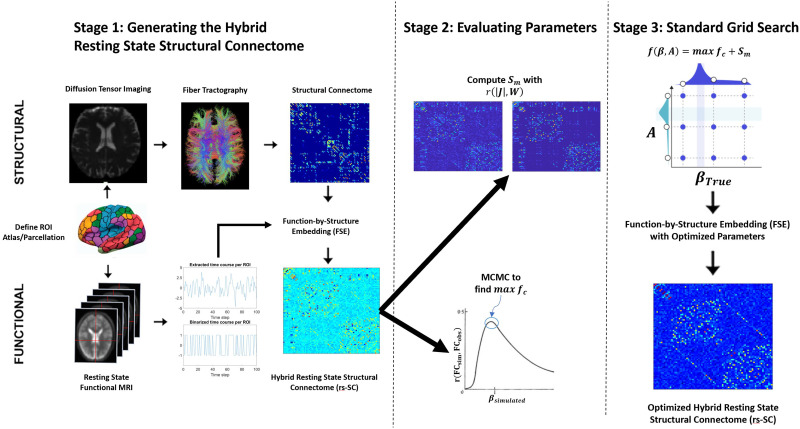
Schematic for the function-by-structure embedding (FSE) and ensuing parameter optimization strategy. The framework for constructing the hybrid resting-state structural connectome using the FSE is based on the principle of maximum entropy. Using a constrained maximum likelihood estimation where structural and functional connectivity are combined, we estimate both an edge strength in the network as well as a sign (±) representing excitatory or inhibitory interactions (Fortel et al., 2019). In Stage 2, two metrics are used to evaluate parameter quality, namely a similarity metric ***S***_***m***_ and the maximum of a functional correlation function *max* ***f***_***c***_. As these values are dependent on parameter choices within the FSE framework, in Stage 3 a grid search is performed to find the optimal values for the tuned parameters that maximize *f*(***β***, ***A***) = *max* ***f***_***c***_ + ***S***_***m***_. These two metrics were computed for each subject individually, to identify the optimal parameters for constructing a hybrid resting-state structural connectome (rs-SC) for each subject.

Here we may construct the gradient ascent procedure with respect to *J*_*i*,*j*_ by computing the partial derivative of the log-pseudolikelihood as the following:

$$\frac{\partial \;\ell }{\partial \;J_{i,j}}=\frac{1}{t_{\max}}\;\sum_{t=1}^{t_{\max}}\beta \left\{s_{i}\left(t\right)s_{j}\left(t\right)-s_{j}\left(t\right)\tanh\left(C_{i}\left(t\right)\right)\right\}-\lambda \left(J_{i,j}-\text{sgn}\left(J_{i,j}\right)W_{i,j}\right),$$ (6)

$$\;\propto \frac{1}{t_{\max}}\;\sum_{t=1}^{t_{\max}}\left\{s_{i}\left(t\right)s_{j}\left(t\right)-s_{j}\left(t\right)\tanh\left(C_{i}\left(t\right)\right)\right\}-A\left(J_{i,j}-\text{sgn}\left(J_{i,j}\right)W_{i,j}\right),$$ (7)

where *A* = $\frac{\lambda }{\beta }$. The updating scheme follows: $J_{i,j}^{n+1}$ = $J_{i,j}^{n}$ + *γ*$\frac{\partial \;\ell }{\partial \;J_{i,j}}$|_*n*_. Here, *n* is the iteration number and *γ* is the learning rate. In this way, the penalty function ensures that the inferred pairwise interaction is scaled relative to the estimated structure of the brain. This process is followed for all 76 subjects as part of first stage in constructing an optimized rs-SC as shown in the schematic of Figure 1. The next stage involves optimization of the two parameters that control scale and convergence, *β* and *A*.

### Determining Parameters for Generating the Optimal Resting-State Structural Connectome

Within the framework for the FSE, parameters that need to be tuned are the constraint scale (parameter *A*) and the convergence parameter *β*. We note two points here: First, in the gradient ascent procedure, the influence of the *β* parameter is primarily in the hyperbolic tangent, which converges to 1 as *β* → ∞ and 0 as *β* → 0. Second, in a similar fashion, the influence of *A* is in the scale of the constraint. Thus, if as *A* → 0, then the model converges to an unconstrained pMEM, and if *A* → ∞, then the constraint will completely dominate the ascent and the system will converge to a pure structural connectome.

**Figure 2.**
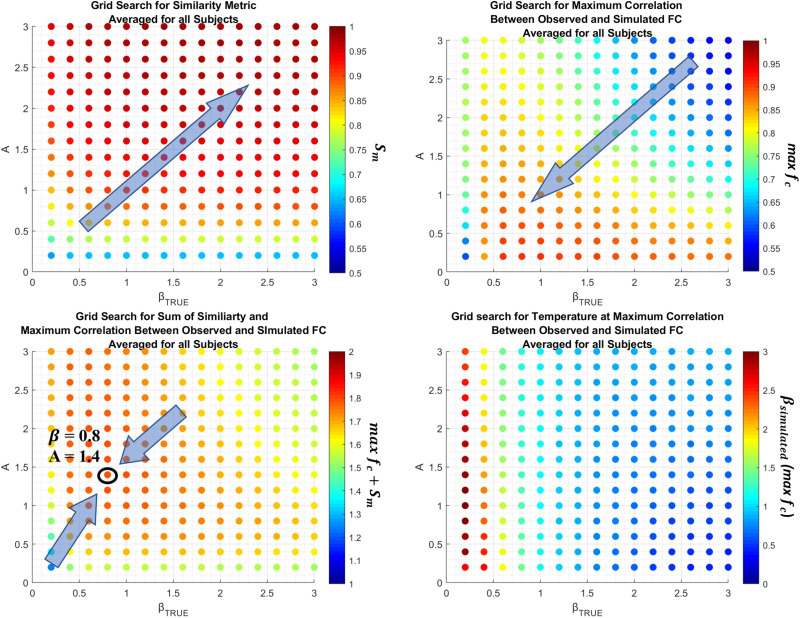
Grid-search parameter optimization for FSE framework. Presented here are example grid-search results based on the proposed optimization strategy as a function of *β*_*True*_ and *A* = $\frac{\lambda }{\beta_{\text{True}}}$. Here, *β*_*True*_ is the value of *β* used in the FSE algorithm. In the top left is the average *S*_*m*_, the correlation *r*(|***J***|, ***W***) used to evaluate the performance of the constraint. The maximum value achieved in this example is *r* = 0.979 when *β*_*True*_, *A* are maximized. In the top right is the average of *max* *f*_*c*_, computed using MCMC simulations for the Ising model as described in the Methods section. The maximum value achieved is *r* = 0.91 for *β*_*True*_ = 0.6, *A* = 0.2. Given the inverse effect of these two metrics, we compute *f*(*β*, *A*) = *max* *f*_*c*_ + *S*_*m*_, identifying a parameter set that maximizes both metrics. Thus, in the grid search the maximum value is achieved at *f*(*β*, *A*) = 1.78 where *β* = 0.8 and *A* = 1.4. Last, in the bottom right is the average *β*_*simulated*_ (*max* *f*_*c*_) during MCMC simulations. We note that as the parameters *β*, *A* → 0, *β*_*simulated*_ (*max* *f*_*c*_) converge to the unconstrained pMEM, and as *β*, *A* → ∞, *β*_*simulated*_ (*max* *f*_*c*_) → 1.

With that in mind, we develop two metrics to evaluate the accuracy and performance of our rs-SC, constructed with our FSE method. First is a similarity metric *S*_*m*_(*β*, *A*) that, as described in the Methods section, is simply the correlation between |rs-SC| and the structural connectome. This metric is used to gauge the quality of the constraint component in the framework. Second, we generate a correlation function *f*_*c*_(*β*, *A*) by simulating the Ising model with Markov chain Monte Carlo (MCMC) simulations (see Methods), computing a Pearson correlation between observed and simulated functional connectivity for all *β*_*simulated*_. As shown in Stage 2 of Figure 1, this results in a bell-like curve where each point represents a correlation value between the observed FC and reconstructed FC for each *β*_*simulated*_. Here we identify the *max* *f*_*c*_(*β*, *A*), which represents the maximum achieved correlation between observed and reconstructed FC for the parameters *β*, *A*. As described in the Methods section, we perform these computations for a range of empirically determined values. The results are presented in Figure 2. As expected, as the parameter values of *β*, *A* increase, the similarity metric *S*_*m*_ evaluating the constraint increases to be almost perfectly correlated with the structural connectome. However, the quality of FC reconstruction, evaluated using *max* *f*_*c*_(*β*, *A*), decreases as the values of *β*, *A* increase. This suggests that, while structural connectivity is important for reconstructing functional dynamics, a purely structural network is limited in the accuracy of FC reconstruction. Hence, using a grid-search technique shown in Stage 3 of Figure 1, we compute *f*(*β*, *A*) = *max* *f*_*c*_ + *S*_*m*_, which intends to take equal weight between the underlying structure of the rs-SC and the accuracy with which it can reconstruct FC. As shown in Figure 2, this grid-search optimization results in the optimal values of *β* = 0.8 and *A* = 1.4 for a single subject (shown as an example). This process is computed individually for each subject, resulting in a unique parameter set for each individual. The range of parameter values is presented in Supplementary Figure 1, noting that the approximate mean value for *A* = 1.75 and *β* = 0.75. Using these parameters, we then reconstruct an optimized rs-SC for each of the 76 subjects to be used in the ensuing analyses.

**Figure 3.**
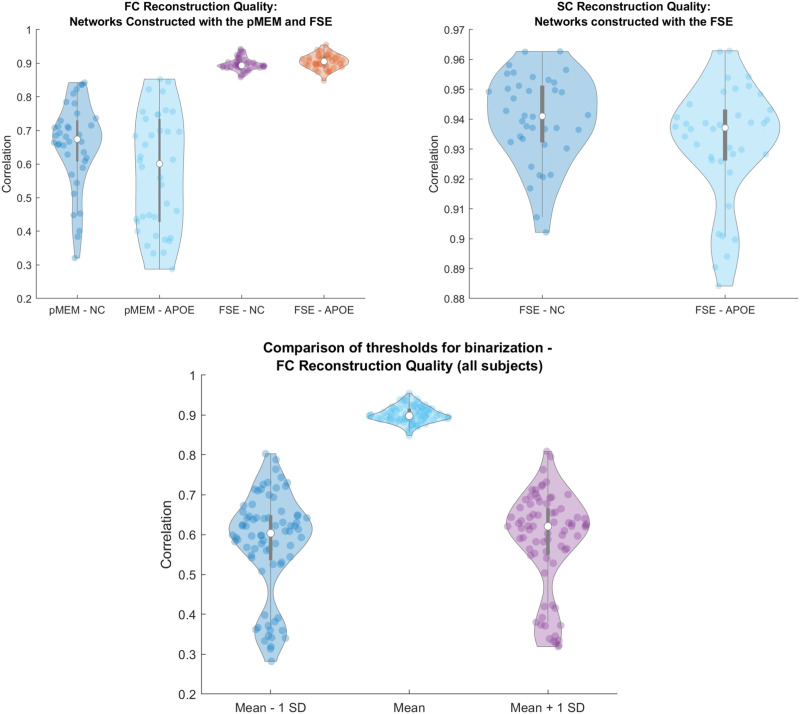
Violin plots evaluating optimal hybrid resting-state connectomes (rs-SC), generated using the FSE framework. The top left plot evaluates the reconstruction quality, that is, the ability of our new network to reconstruct traditional FC correlation patterns. We use the network estimated using an unconstrained pMEM as a control for comparison. Using these networks, we identify a *max* *f*_*c*_ value, representing the maximum correlation between the observed FC and reconstructed FC using MCMC simulations of the Ising model as described in the Methods section. Presented here are results for the noncarrier (NC) and APOE groups based on the two estimation techniques. The median *max* *f*_*c*_ for (NC, APOE) using the pMEM is (0.67, 0.60) with a range of {(0.84, 0.32), (0.85, 0.28)}. The 95% CI for the NC group is 0.66 ± 0.036 and 0.6 ± 0.044 for the APOE group. Using the FSE, the median correlation for (NC, APOE) is (0.89, 0.90) with a range of {(0.94, 0.85), (0.94, 0.84)}. The 95% CI for the NC group is 0.89 ± 0.015 and 0.87 ± 0.021 for the APOE group. Last, given that the FSE framework relies on the structural connectivity as a constraint on the network estimation, we evaluate the quality of the constraint using the similarity metric *S*_*m*_ described in the Methods section. In the top right plot, the median *S*_*m*_ for both groups is 0.94 with a range of (0.96, 0.90) in the NC group and (0.96, 0.88) in the APOE group. Further, the 95% CI for the NC group is 0.93 ± 0.0017 and 0.93 ± 0.0003 in the APOE group. These results suggest strong and consistent performance of the structurally informed rs-SC in reconstructing functional dynamics for both groups, as well as constraint on the estimated network. Last, the bottom plot presents an evaluation of binarization thresholds using the *max* *f*_*c*_ value, representing the maximum correlation between the observed FC and reconstructed FC using MCMC simulations of the Ising model. The median value when binarizing about zero is 0.89, while the median value is 0.62 and 0.60 for ±1 *SD*, respectively. These results reveal outliers when binarizing the time series data using a value other than zero. This is most likely due to data quality questions related to fMRI processing resulting in time series with up to 40% of the TRs excluded due to imaging artifacts for some subjects.

Using this optimization process, we further tested different binarization strategies for the z-scored time series data to identify the optimal thresholding parameter. Here we tested 0, and ±1 *SD*. We used *max* *f*_*c*_(*β*, *A*) as a measure of performance to determine whether one threshold results in better FC reconstruction quality. The results are presented in Figure 3, noting that using zero as the threshold results in more consistent and accurate quality. We note the existence of a handful of outliers when binarizing ±1 *SD*. This is in part due to processing steps of the observed BOLD time series, where upwards of 40% of the TRs were excluded due to imaging artifacts for some subjects. As with all inference-based methods, accuracy of estimations increases or decreases with the amount of observed data. Future studies using this methodology will focus on cohorts with more consistent time series data across subjects.

### Evaluating the Quality and Performance of the Resting-State Structural Connectome (rs-SC)

With the now optimized rs-SC it is important to compare this combined structure-function network with our control (or null model) pMEM-based interaction network, and the traditional Pearson-correlated FC. To do this we first perform MCMC simulations of the Ising model with 64,000 runs (*N* × *N* × 10; *N* = 80 ROIs) over a range of *β*_*simulated*_ (see the Methods section). In this we may compute the *max* *f*_*c*_ correlation results for each participant using the pMEM-based network and FSE-based networks (rs-SC), comparing the performance of both in reconstructing observed functional connectivity. In evaluating the results, we also separate our 76 subjects into two groups of noncarriers (NC) and carriers (APOE) to determine whether there are any within-group performance differences. The results of these simulations are presented in Figure 3, with the violin plot showing the median and range of *max* *f*_*c*_ correlation values. The median *max* *f*_*c*_ for (NC, APOE) using the pMEM-based interaction network is (0.67, 0.60) with a range of {(0.84, 0.32), (0.85, 0.28)}. The 95% CI for the NC group is 0.66 ± 0.036 and 0.6 ± 0.044 for the APOE group. Using the FSE-based rs-SC, the median correlation for (NC, APOE) is (0.89, 0.90) with a range of {(0.94, 0.85), (0.94, 0.84)}. The 95% CI for the NC group is 0.89 ± 0.015 and 0.87 ± 0.021 for the APOE group. Based on comparison with the pMEM-based network, the FSE-based network results in a more consistent and accurate reconstruction of FC for both the NC and the APOE groups. Further, we evaluate the quality of our constraint under the optimized parameters *β*, *A* for all subjects. Violin plots for both NC and APOE groups, evaluating the similarity metric *S*_*m*_, are presented in Figure 3. The median *S*_*m*_ for both groups is 0.94 with a range of (0.96, 0.90) in the NC group and (0.96, 0.88) in the APOE group. Further, the 95% CI for the NC group is 0.93 ± 0.0017 and 0.93 ± 0.0003 in the APOE group. These results suggest strong and consistent performance of the structurally informed rs-SC in reconstructing functional dynamics for both groups, as well as constraint on the estimated network.

**Figure 4.**
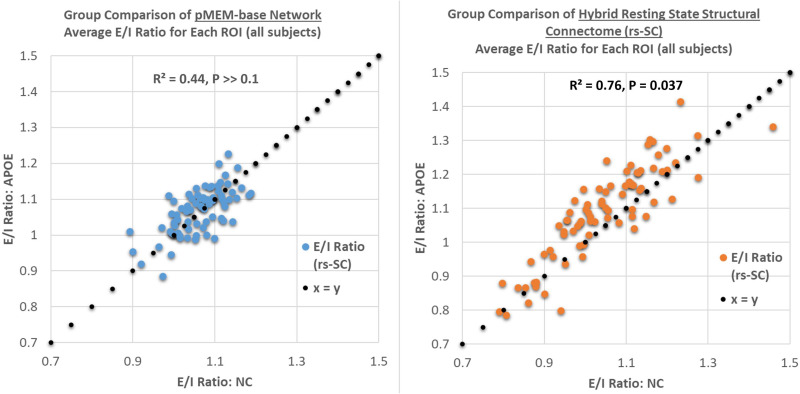
Group comparison of the excitation-inhibition ratio for each brain region based on the unconstrained pairwise maximum entropy model and the function-by-structure embedding. As described in the Methods section, the E/I ratio is simply the sum of positive edges divided by the sum of negative edges for each ROI. Here, we present a plot comparing the E/I ratio between the NC and APOE groups using the pMEM-based network and our rs-SC network, computed and averaged at the ROI level. This results in a weak association with *R*^2^ = 0.44 for for the pMEM-based network, and *R*^2^ = 0.76 for the rs-SC network, with paired *t* tests across all ROIs resulting in *P* >> 0.1 and *P* = 0.037, respectively. This suggests no statistically significant differences in E/I balance when using the unconstrained model; however, there is a statistically significant difference in the two groups when using our structurally informed model. We note that numerically, an increase in group-averaged E/I ratio would move a point (representing one ROI) above the *x* = *y* reference line, suggesting a shift in E/I balance towards hyperexcitation. A tabular version of these results is included in the Supporting Information.

**Figure 5.**
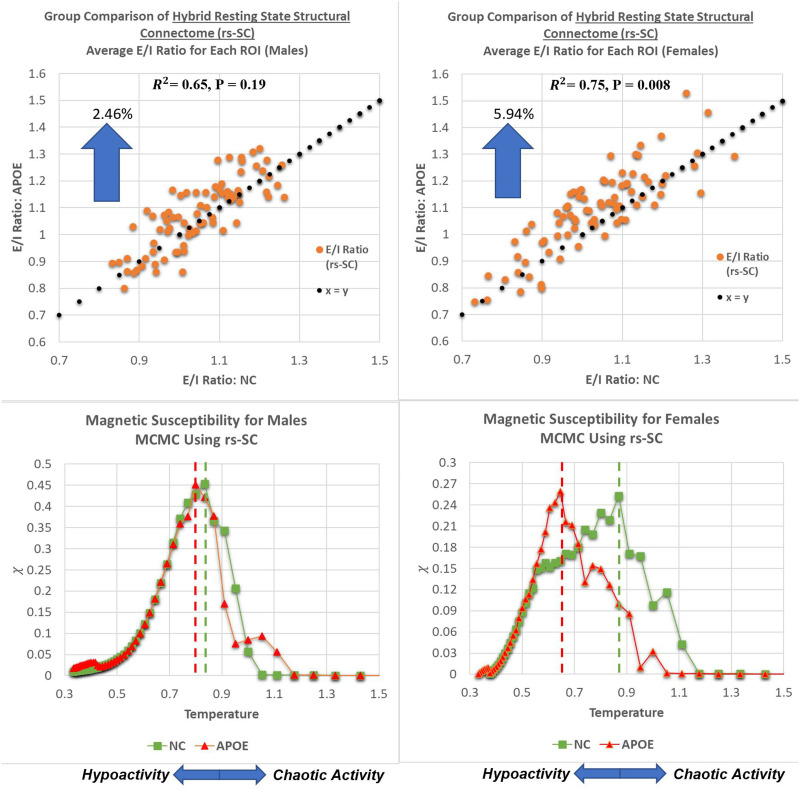
Gender-based comparison of critical behavior and E/I balance. As described in the Methods section, the E/I ratio is simply the sum of positive edges divided by the sum of negative edges for each ROI. In the top panels, we present plots comparing the E/I ratios between the NC and APOE groups for males and females, computed and averaged at the ROI level. This results in a strong association with *R*^2^ = 0.65 for males, and *R*^2^ = 0.75 for females with paired *t* tests across all ROIs resulting in *P* = 0.19 for males, and *P* = 0.008 for females. This suggests no statistically significant differences in E/I balance for males; however, there is a statistically significant difference for females. We note that numerically, an increase in group-averaged E/I ratio would move a point (representing one ROI) above the *x* = *y* reference line, suggesting a shift in E/I balance towards hyperexcitation with increased risk of chaotic activity. Thus, for each ROI, we can quantify the shift in E/I balance by computing *delta* = 1 − $\frac{{\left(E/I\right)}_{NC}}{{\left(E/I\right)}_{\text{APOE}}}$ to evaluate the average change between NC and APOE groups; this yields a shift of 5.94% in the female group between carriers and noncarriers, while in the male group it is 2.46% (approximately 2.4× difference between sexes). A tabular version of these results is included in the Supporting Information.

Last, in previous studies no group differences could be identified between NC and APOE groups using traditional FC measures (Fortel et al., 2019; Korthauer et al., 2018). Here, we test whether an excitation-inhibition (E/I) ratio may be used to differentiate between the rs-SC network as well as the pMEM-derived interaction network. As defined in the Methods section, the E/I ratio is simply the sum of the positive edges, divided by the sum of negative edges (computed at either the whole-brain or ROI level). For both networks, the E/I ratio is computed for all ROIs and averaged for the NC and APOE groups. In Figure 4, the scatterplots display a weak association between the average E/I ratio for all ROIs between the NC and APOE groups using the pMEM-derived network with *R*^2^ = 0.44, and a paired *t* test across all ROIs results in *P* >> 0.1, suggesting no statistically significant group differences in the pMEM-based networks. Conversely, performing a similar computation of the E/I ratio on the rs-SC networks results in a strong association between NC and APOE groups, with *R*^2^ = 0.76, as well as a notable shift observed for all ROIs (i.e., globally). A paired *t* test between the groups results in *P* = 0.037, suggesting a statistically significant group difference in the rs-SC networks between NC and APOE groups, as evaluated with the E/I ratio that cannot be identified with the unconstrained model. In sum, the results presented in this section indicate that the novel rs-SC network constructed with the FSE framework can not only describe structural and functional dynamics, but also probe brain dynamics that may not be captured using a similar unconstrained methodology. Last, we compare the E/I ratio in a group comparison of males and females (NC versus APOE) and present the results in the scatterplot of Figure 5. Both males and females exhibit a positive association between groups with *R*^2^ = 0.65 and *R*^2^ = 0.75, respectively; however, only the female group has a statistically significant group difference with *P* = 0.008. Here, we perform a calculation on group difference by computing *delta* = 1 − $\frac{{\left(E/I\right)}_{NC}}{{\left(E/I\right)}_{\text{APOE}}}$ to evaluate the average change between NC and APOE groups. The males have an average increase of 2.4% (averaged across all ROIs), while the females have an average increase of 5.9% in E/I ratio (approximately 2.4x higher than the male group). The raw values for each brain region are shared in Supplementary Table 1 for reference.

### Criticality and Hyperexcitation in Female APOE-ε4 Carriers

In this study, our subjects are separated into two age- and sex-matched groups (NC and APOE). One aspect of the link between APOE-ε4 and AD that has often been overlooked is that females with at least one ε4 allele are four times more likely to develop AD than males (Bretsky et al., 1999; Jack et al., 2015; Payami et al., 1994). Thus, we use our framework to evaluate not just group differences in criticality, but sex differences as well (22F/16M in each group). As previously mentioned, the brain criticality hypothesis suggests that neural networks self-organize into a unique configuration between order and disorder. In the context of statistical physics and the Ising model, this unique configuration occurs at some critical point (*β*_*critical*_). Here, we again utilized MCMC simulations to generate a series of state configurations (±1) resulting in an *N* × *t* matrix, where *N* = 80 ROIs, and *t* = 100,000 runs (see Methods). In the previous section we used these states to compute a correlation between brain regions; however, in this case we will evaluate the critical dynamics elucidated from the rs-SC networks. Specifically, we are interested in the phase transitions based on the positive edges of the networks. The Ising model can be modified to model spin-glass behavior (full signed network); however, this can lead to “frustration” in the simulations. Frustration describes a scenario in which it is impossible to simultaneously minimize all the terms in the Hamiltonian. As a result, this generally leads to complex energy landscapes with many local minima. At low *β*, the system can get stuck in the local minima without ever reaching a true equilibrium. In future work we can investigate thermodynamic properties using the full signed network (spin-glass), but here we proceed in evaluating the ferromagnetic phase transitions.

For each *β*, we compute the order parameter (magnetization) and the variance (susceptibility) with respect to *β*_*simulated*_. Here, we again compute average values over NC and APOE groups to investigate potential group differences in critical behavior. As described in the methods, β is the inverse temperature (*T*) parameter used in the Boltzmann distribution, and thus when simulating dynamics to identify phase transitions, we interpret temperature as a tolerance of the system when increased randomness is introduced. Performing Monte Carlo simulations of the Ising model using our hybrid network for a range of temperatures is used to identify a critical point, such that the system transitions from a hypoactive regime to a chaotic regime. Hence, the critical temperature is a measure of how much tolerance the system has to increased perturbations. We present the phase diagrams for susceptibility in Figure 5 for males and females, highlighting *T*_*critical*_ for both groups (evaluated by the peak of susceptibility). It should be noted that *β* was simulated from 0.2 to 3.0 at increments of 0.05 (then plotted against *T* = $\frac{1}{\beta }$). We identified a more pronounced deviation between NC and APOE females with *T*_*critical*_ = 0.65 for the female APOE group as compared with *T*_*critical*_ = 0.87 in the NC group. Conversely, *T*_*critical*_ = 0.80 in the male APOE group as compared with *T*_*critical*_ = 0.83 in the NC group. This suggests that the critical dynamics within the male group between NC and APOE are more similar in nature than the dynamics observed within the female group between NC and APOE. A lower critical temperature in the female carrier group suggests a lower tolerance to network dysfunction as a result of an increase in excitatory interactions, increasing vulnerability to chaotic activity. In sum, these results suggest that there is a link between brain criticality and excitation-inhibition balance that can be identified via our new connectome, demonstrating a disruption to this balance in APOE carriers (with a larger effect in females).

Further, presented here are plots demonstrating a global evaluation of critical brain dynamics. In the bottom panels, ferromagnetic susceptibility is shown for males and females, with the dashed lines representing the critical point *T*_*critical*_ = *T*_*simulated*_(*max* **χ**). These charts demonstrate a more pronounced deviation between NC and APOE females with *T*_*critical*_ = 0.65 for the female APOE group as compared with *T*_*critical*_ = 0.87 in the NC group. Conversely, *T*_*critical*_ = 0.80 in the male APOE group as compared with *T*_*critical*_ = 0.83 in the NC group. This suggests that as the E/I balance shifts at global scale, the critical point also decreases because of an increase in excitatory interactions. As described in the Methods section, a lower critical temperature indicates a lower tolerance to network dysfunction, increasing vulnerability to chaotic activity.

## DISCUSSION

Using a constrained maximum entropy model for our function-by-structure embedding (FSE), we have developed here a novel resting-state structural connectome (rs-SC), unifying connectome-level structure and function into a new spatiotemporal network. We constructed rs-SC networks for 76 cognitively intact participants with a grid-search parameter optimization scheme. Hence, we demonstrate two important results: First, the underlying structure of the rs-SC is as expected, strongly correlated with the empirical structural connectome (*r* > 0.9) due to it being used as a constraint in the FSE framework. Second, and more importantly, we demonstrated that it is possible to model the resting-state functional connectome based on a model of spin products, accounting for indirect or higher order structural connectivity. We acknowledge that when Ising dynamics are used to model neural firing patterns, these activations may amount to the collective behavior of a few neurons, and at the macro level of fMRI imaging used in this study each voxel may be providing information as a result of thousands of interacting neurons. However, simulation and empirical studies have demonstrated that increases in excitatory neuronal activity amplify oscillations associated with the transient BOLD response, while increasing inhibitory activity evokes an overall decrease in the BOLD signal (Aksenov et al., 2019; Krishnan et al., 2018; Sotero & Trujillo-Barreto, 2007; Sten et al., 2017). By grounding our macroscale methodology with models of microscale dynamics, we bridge the gap between the two, hereby inferring the nature (excitatory or inhibitory) of structural connectivity at rest. Further, the rs-SC can be used to simulate functional dynamics using Monte Carlo simulations, reconstructing traditional functional correlation patterns (*r*_*avg*_ = 0.9). Beyond model quality and performance, we have also demonstrated that our rs-SC can distinguish between female noncarriers and APOE-ε4 carriers (age- and sex-matched) using our excitation-inhibition (E/I) ratio. Our results demonstrate that modeling with the rs-SC reveals a global shift of E/I balance for the APOE-ε4 carrier group. Given that APOE-ε4 carriers are at an elevated risk for AD, the observed shift in E/I balance in this sample may be a result of disease pathology. In many studies of AD, one critical feature that is often overlooked is that females with at least one ε4 allele are four times more likely to develop AD than are males (Jack et al., 2015). A comparison of group-averaged E/I ratio at the ROI level for each sex using the rs-SC (with new optimization strategy) yielded a global shift in E/I balance towards hyperexcitation, in line with our previous work (Fortel et al., 2020) and prior studies on sex differences related to the APOE genotype (Aboud et al., 2013; Bi et al., 2020; Jiménez-Balado & Eich, 2021; Leung et al., 2012). In future work, we may investigate in depth the relationship of our hybrid connectome with traditional measures of structural and functional connectivity in a larger cohort (with increased age range), to investigate known sex differences and further evaluate our method.

Further, in this study, we observe significant differences in critical behavior between a group of cognitively intact individuals with a genetic predisposition for late onset AD as compared with age- and sex-matched noncarriers. Traditional structural and functional connectivity based on BOLD correlations were unable to separate the two groups (Fortel et al., 2020; Korthauer et al., 2018). These results suggest that using a multimodal framework to unify structure and function can reveal underlying patters in brain dynamics that would otherwise not be captured using traditional methods. Further, we endeavored to identify a link between E/I balance and criticality. As a result of increased positive interactions (increased deviation from an E/I balance) in the hybrid connectome, simulations of brain dynamics using Monte Carlo simulations revealed a shift in criticality for female carriers compared with noncarriers of APOE-ε4 that may suggest an increased vulnerability to AD neuropathology in female APOE-ε4 carriers. We describe the critical temperature as a measure of tolerance in our modeled system that we simulate in dynamical regimes spanning from highly ordered (i.e., hypoactive) to highly disordered. This is in line with studies of preclinical neural models that have shown that networks operating at criticality exhibit an E/I balance as compared with networks that have been over excited or overinhibited by a controlled chemical stimulus (Heiney et al., 2019; Shew et al., 2011). In fact, many of the in vivo studies that have investigated the criticality hypothesis and excitation-inhibition balance in neurodegenerative disorders have relied on electroencephalography (EEG) or magnetoencephalography (MEG) recordings (Bruining et al., 2020; Montez et al., 2009; Rajkumar et al., 2021; Stam et al., 2005), which have inherent challenges with spatial resolution. By defining our activity states using both structural and functional connectivity together, we are capable of analyzing patterns of activity across both temporal and spatial scales, thereby improving the network inference and mitigating many challenges observed in unimodal and traditional analyses.

The results presented herein regarding E/I balance, criticality, and the APOE-ε4 genotype also coincide with the current understanding of the microscale mechanisms underlying AD pathology. A recent review article by Najm and colleagues explored the relationship among APOE-ε4, loss of GABAergic interneurons, and dysfunctional brain networks in the context of AD (Najm et al., 2019). In short, neurons responding to different factors (e.g., normal aging, injury, or stress) break down APOE-ε4 proteins and produce fragments that trigger phosphorylation of tau; this in turn disrupts mitochondrial function, leading to cell death. Destruction of inhibitory neurons in this way can alter network activity and produce hyperexcitability in neural circuits long before clinically identifiable symptoms arise. This may help explain the known associations of APOE-ε4 with memory deficits and severe epilepsy. Indeed, several in vitro and preclinical in vivo studies (cited by Najm et al., 2019) have demonstrated that intracellular APOE-ε4 is toxic to GABAergic interneurons, particularly in the hippocampus.

Moreover, other authors have recently suggested that neuronal hyperexcitability may be considered to be both a causal factor and a risk factor in the disease progression, even in the preclinical phase (Hijazi et al., 2020; Paterno et al., 2020; Tok et al., 2021). While significant structural and functional degeneration is well established in AD (DeTure & Dickson, 2019), our framework incorporates both structural and functional connectivity in order to provide a new multimodal perspective of connectome-level interactions in a preclinical group of individuals predisposed to AD. We acknowledge that our methodology is limited to insights that may be gained from macroscale BOLD activity as opposed to direct measurements of neuronal processes. That said, we reached a similar conclusion to independent studies of underlying neural mechanisms in AD: Individuals with the APOE-ε4 allele (females in particular) have a higher risk of neurodegeneration due to an increase of excitatory activity in neural circuits (Jiménez-Balado & Eich, 2021; Koutsodendris et al., 2022; Y. Li et al., 2016).

We note several limitations of this study. First, this study investigated only a small cross section of healthy middle-aged individuals at increased risk of developing AD. Further, the parcellation used in the processing used an atlas with 80 brain regions, which may be considered too coarse. Additional research with a longitudinal cohort and higher resolution parcellation would help improve the generalizability of results, providing important validation regarding within-subject variability, as well as broadening our understanding of longitudinal alterations in brain dynamics. Second, when interpreted as a strictly nodal property, excitation-inhibition balance may be best measured at a regional level using FDG PET or phosphorous imaging. However, as conceptualized in this study, the concept of E/I balance may directly relate to this notion of “criticality” in brain dynamics. Further, in this group of participants, measurements of well-known biomarkers of Aβ and tau were not included in the protocol, and thus we could not add this layer of validation. Future studies comparing additional imaging modalities and biomarkers for validation and correlation purposes may be used to strengthen the results and methodology presented in this study (in addition to more state-of-the-art diffusion tensor imaging and fMRI imaging protocols). Further, in this study as we are working with resting-state data processed with global signal regression (accounting for background and nonneural physiological noise), we model the BOLD activity assuming no external influences; future work can incorporate external influences in the framework to account for different interference scenarios.

It remains unclear whether the difference in criticality observed between the NC and APOE groups is because the NC group (on average) contains more inhibitory interactions or whether the APOE group has more excitatory interactions. Since we do not identify directionality in this study, this question is left for future work. Additionally, we have not performed an assessment herein on the potential relationships between traditional structural and functional connectivity measures, and metrics obtained with our rs-SC. This may be explored in detail with future investigations. Further, at the coarser spatial scale of human fMRI, there is evidence that the strength of functional connectivity between regions is greatest for region pairs separated by short physical distance and that connectivity strength decays rapidly as the Euclidean distance between brain regions increases (Alexander-Bloch et al., 2013). Likewise, the extent of white matter tract connectivity as measured with diffusion imaging also decays with distance. However, the inverse relationship between fMRI-based connectivity and distance is significant even after controlling for the strong association between anatomical connectivity and functional connectivity (Honey et al., 2009). In the future, the role of distance related to excitatory and inhibitory interactions should be explored in greater depth. Further utilizing thermodynamic principles, it should be investigated whether the rs-SC decays algebraically with a distance *d* (i.e., ***J***(*d*) ∝ *d*^−*α*^) as well as what, if any, effect this distance decay would have on critical brain dynamics. Given the complex inner workings of the brain, it is entirely plausible that dynamics between brain regions at or near criticality rely on a balance between long- and short-range interactions. Again, this suggests that functional brain dynamics are governed by the underlying structure of the networks. Thus, after decades of research studying the brain’s individual components, from neurons to neuronal ensembles and large-scale brain regions, conclusive evidence demonstrates the need for maps and models that incorporate interactions among these components in order to better understand the brain’s ensemble dynamics, circuit function, and emergent behavior.

## MATERIALS AND METHODS

### Participants and MRI Data Acquisition

The cohort used in this work has been described in a previous study (Korthauer et al., 2018). Participants (*N* = 76; all Caucasian) were selected based on APOE genotype from a larger sample of 150 adults aged 40–60 (age = 49.9 ± 6.0 in years; 60 men). The University of Wisconsin–Madison Biotechnology Center conducted the sequencing of the single nucleotide polymorphisms (SNPs; rs7412, rs429358) making up the common ε2, ε3, and ε4 APOE genotypes. Thirty-eight individuals out of the larger sample were APOE-ε4 carriers (either ε3/ε4 or ε4/ε4). Hence, a subset of noncarriers (ε3/ε3 or e2/ε3) were age- and sex-matched, creating equal groups (*N* = 38, 22 female) of carriers (APOE) and noncarriers (NC). The following exclusion criteria were used: (a) self-reported cognitive or memory complaints; (b) Mini–Mental State Exam (MMSE; Folstein et al., 1975) score ≤ 24; (c) Mattis Dementia Rating Scale Second Edition (DRS-2; Johnson-Greene, 2004) score ≤ 135; (d) Geriatric Depression Scale (GDS; Yesavage et al., 1982) > 10; (e) history of central nervous system disease (e.g., dementia, stroke, Parkinson’s disease, epilepsy, other neurological disease); (f) history of severe cardiac disease (e.g., myocardial infarction, coronary bypass surgery, angioplasty); (g) history of metastatic cancer; (h) history of serious psychiatric disorder or substance use disorder; and (i) any contraindication to MRI. MRI imaging was conducted on a GE Signa 3T scanner (Waukesha, WI) with quad split quadrature transmit/receive head coil. All participants provided written informed consent, and were compensated financially for their participation; the imaging collection was carried out in accordance with the guidelines set by the institutional review boards of the University of Wisconsin–Milwaukee and Medical College of Wisconsin (Korthauer et al., 2018). Demographic characteristics and screening measures for each group are presented in Table 1.

**Table 1.** Demographic characteristics and screening measures. Values represent *M*(*SD*). DRS-2: Mattis Dementia Rating Scale-2. MMSE: Mini–Mental State Examination. GDS: Geriatric Depression Scale.

	*ε4 carriers (N = 38)*	*non-ε4 carriers (N = 38)*
Age (years)	50.8 (0.99)	50.9 (0.99)
Sex (M:F)	16:22	16:22
Education (years)	15.4 (2.5)	15.2 (2.4)
DRS-2 (total)	139.9 (2.3)	139.9 (2.3)
MMSE (total)	28.5 (1.1)	28.8 (1.3)
GDS (total)	1.8 (2.3)	2.4 (2.7)

All participants were screened for any contraindications to MRI. Imaging sessions lasted 75 min. To determine the structural and functional connectivity maps, multimodal imaging (including T1-weighted MRI, resting-state fMRI, and diffusion weighted MRI) was performed. For structural MRI imaging, a “spoiled-grass” (SPGR) sequence (axial acquisition: TR = 35 ms, TE = 5 ms, flip angle = 45°, matrix = 256 × 256, FOV = 24 cm, NEX = 1) was obtained, followed by a T2*-weighted functional scan with an echo-planar pulse imaging (EPI) sequence (28 axial slices, 20 × 20 cm^2^ FOV, 64 × 64 matrix, 3.125 mm × 3.125 mm × 4 mm voxels, TE = 40 ms, TR = 2,000 ms). The 8-min rs-fMRI scan was acquired while participants were under task-free conditions (i.e., resting state). Additionally, a 3-min, 30-s diffusion tensor imaging sequence was acquired with a spin echo single shot, echo-planar imaging sequence with sensitivity (SENSE = 2.5) encoding (2.2 mm isotropic voxels, 212 × 212 mm FOV, 96 × 96 acquired matrix), TR/TE = 6,338/69 ms, 60 slices for whole-brain coverage. Diffusion gradients were applied along 32 noncollinear directions at a b-factor of 700 s/mm^2^, including one minimally weighted image with b = 0 s/mm^2^.

### Processing of fMRI and Diffusion Tensor Imaging

Preprocessing of rs-fMRI images was performed using Analysis of Functional NeuroImages (AFNI; Cox, 1996) and FMRIB Software Library (FSL; Smith et al., 2004) based on the rs-fMRI preprocessing pipeline from the Human Connectome Project (HCP; Smith et al., 2013). Detailed processing information steps can be found in prior work (Korthauer et al., 2018). Diffusion tensor imaging data processing was carried out with the FSL. The B0 image was skull-stripped using the brain extraction tool (Smith, 2002), with the resulting mask applied to the other images. Eddy current-induced distortions and subject movements were corrected using FSL’s *eddy* tool (Andersson & Sotiropoulos, 2016). A probability distribution for fiber direction was generated at each voxel using BEDPOSTX (Behrens et al., 2007; Behrens et al., 2003), which was then used in probabilistic tractography. For individual subjects, FreeSurfer cortical parcellation and subcortical segmentation was used, defining the 80 ROIs (Dale et al., 1999; Fischl et al., 2002; Fischl et al., 2004). Affine registration with 6 degrees of freedom using FLIRT registered the ROIs to MNI and diffusion space (Jenkinson et al., 2002). For each ROI, the mean time course from the BOLD signal was extracted using global signal regression (GSR) from the preprocessed rs-fMRI data prior to constructing the functional connectivity matrix. The resulting zero-mean time courses for each ROI were then correlated using Pearson correlations to generate a traditional functional connectivity matrix. Probabilistic tractography was performed between pairs of ROIs using Probtrackx for estimating the structural connectivity. The resulting matrix was then further normalized by dividing each matrix row by the way-total for its corresponding seed ROI (Behrens et al., 2007; Behrens et al., 2003).

### The Unconstrained Pairwise Maximum Entropy Model (pMEM)

This maximum entropy approach provides a way of quantifying the goodness of fit in models that include varying degrees of correlations (Schneidman et al., 2006). At a microscale level for example, a first-order model seeks to fit only the average firing rate of all neurons recorded in the ensemble. A second-order model would seek to fit the average firing rate and all pairwise correlations, with an *n*th-order model fitting all correlations up to and including those between all *n*-tuples of neurons in the ensemble. At macroscale, this amounts to fitting the average BOLD activation rate of a brain region and all pairwise correlations. Here, the observed bold activation rate is determined through a binarization of the BOLD time course. Thus, we construct unbiased predictions for the probabilities of functional brain states by fitting a pairwise maximum entropy model (pMEM). Here, in estimating the probability distribution, it is necessary to use the distribution that maximizes the uncertainty (e.g., entropy). To fit the pMEM, we must tune the first- and second-order interaction parameters between ROIs such that the predicted activation and coactivation rates match the observed data (the BOLD time series). An accurately fitted pMEM suggests that patterns of functional activity can be estimated from each ROI’s independent activation rate combined with the joint activation rates. Thus, the pMEM represents a model of fMRI BOLD dynamics as a probabilistic process defined by underlying pairwise relationships between ROIs. In constructing this model, we leverage the Ising model, a special case of a Markov random field in which each ROI can exhibit two possible states *s* = ± 1. In this work, we first convert our BOLD time series to z-scores, ensuring that our BOLD date is represented as zero-mean with unitary variance, without altering the correlations between brain regions. As maximum entropy models of neural activity are developed based on Ising dynamics, studies investigating pairwise interactions using BOLD time course data are binarized to define activation states (either +1 for active, or −1 for inactive) in both simulated and empirical fMRI-based studies (Ashourvan et al., 2021; Cofré et al., 2019; Ezaki et al., 2020; Ezaki et al., 2017; Gu et al., 2018; Nghiem et al., 2018; Niu et al., 2019; Watanabe et al., 2013). We will show how the binarization strategy may be validated using Monte Carlo simulations, whereby we use the inferred interaction networks to reconstruct functional correlations. Our results will also show that for our network construction methodology, binarizing the z-scored time series at zero provides better inference of functional interactions than ±1 *SD*.

We first begin by modeling the neural system using an energy-based formulation, namely the Hamiltonian, as follows:

$$H\left(s\right)=-\sum_{<i\;j>}J_{i,j}s_{i}s_{j},\;\text{where}\;i,j\in \left[1,2,\ldots ,k\right]$$ (8)

Here, the spin configuration *s* is defined as the column vector **s** = [s_1_, s_2_, …*s_k_*]^*T*^, *k* is the number of regions, *s*_*i*_ and *s*_*j*_ are the spin states of region *i* and *j*, and *J*_*i*,*j*_ represents a pairwise interaction between ROIs. Conceptually, if two regions are co-active or co-inactive, the pairwise interaction is likely positive (excitatory), and if one region is active while the other is inactive, the pairwise interaction is likely negative (inhibitory). Here we assume that there is no external influence (i.e., resting state). Further, unless otherwise stated, the summations in this manuscript are for *i* < *j* to avoid double counting and exclude self-connections. The probability of observing a specific configuration is given as the following Boltzmann distribution:

$$\text{Pr}\left(s\right)=\exp\left(-\beta H\left(s\right)\right)/Z,$$ (9)

where *β* is the inverse temperature, and *Z* is the partition function: *Z* = ∑_***s***_ exp(−*βH*(***s***)).

The summation in the partition function is over all possible configurations of states. Similar to other studies fitting pairwise models to neuronal firing data, a gradient ascent updating scheme is used (Watanabe et al., 2013; Yeh et al., 2010). Estimating a parameter set that minimizes the Kullback-Leibler (K-L) divergence between modeled and observed probability distributions is equivalent to maximizing a log likelihood of the observed data (the empirical BOLD time series). We note that a brute-force application of the maximum likelihood estimation requires heavy computational costs with calculations over all 2^*N*^ possible spin configurations for the partition function (Nguyen et al., 2017). To overcome the intractability of the partition function *Z*, we utilize a pseudolikelihood estimation method (Ezaki et al., 2017). Pseudolikelihood estimation has been shown to converge to a maximum likelihood estimator for large sample sizes (Besag, 1975).

The optimal interaction matrix ***J*** can thus be derived by maximizing the pseudolikelihood function (Besag, 1975, 1977):

$${𝓛}_{\text{peusdo}}\left(J,\beta \right)=\prod_{t=1}^{t_{\max}}\prod_{i=1}^{k}\text{Pr}\left(s_{i}\left(t\right)|J,\beta ,s_{-i\left(t\right)}\right).$$ (10)

Pseudolikelihood substitutes the probability of observing the state vector **s**(t) by the product of the conditional probability $\tilde{p}$ = *Pr*(*s*_*i*_(*t*)|***J***, *β*, ***s***_−*i*(*t*)_) of observing a single element *s*_*i*_(*t*) while all the other elements, denoted ***s***_−*i*(*t*)_, are fixed. Thus, we maximize the following log-pseudolikelihood function as the following:

$$\ell \left(J,\beta \right)=\frac{1}{t_{\max}}\text{ln}{𝓛}_{\text{pseudo}}\left(J,\beta \right)$$ (11)

$$\;=\frac{1}{t_{\max}}\;\sum_{t=1}^{t_{\max}}\sum_{i=1}^{N}\ln\left(\frac{\exp\left(\beta \sum_{k=1}^{N}J_{i,k}s_{i}\left(t\right)s_{k}\left(t\right)\right)}{\exp\left(\beta \sum_{k=1}^{N}J_{i,k}s_{k}\left(t\right)\right)+\exp\left(-\beta \sum_{k=1}^{N}J_{i,k}s_{k}\left(t\right)\right)}\right).$$ (12)

This probability distribution is derived based on the Boltzmann distribution under pseudolikelihood conditions. The numerator describes the energy of the system, while the denominator is the sum of all possible energies. Hence, there are only two terms in the denominator, one positive and one negative since *s*_*i*_(*t*) is binary. The likelihood function may be simplified further by setting *C*_*i*_(*t*) = *β* $\sum_{m=1}^{k}$ *J*_*i*,*m*_*s*_*m*_(*t*), resulting in the following:

$$\ell \left(J,\beta \right)=\frac{1}{t_{\max}}\;\sum_{t=1}^{t_{\max}}\sum_{i=1}^{N}\;C_{i}\left(t\right)s_{i}\left(t\right)-\ln\left(\text{exp}\left(C_{i}\left(t\right)\right)+\text{exp}\left(-C_{i}\left(t\right)\right)\right).$$ (13)

The gradient ascent procedure can now be constructed with respect to *J*_*i*,*j*_ by computing the partial derivative of the log-pseudolikelihood as the following:

$$\frac{\partial \;\ell }{\partial \;J_{i,j}}=\frac{1}{t_{\max}}\;\sum_{t=1}^{t_{\max}}\beta \left\{s_{i}\left(t\right)s_{j}\left(t\right)-s_{j}\left(t\right)\tanh\left(C_{i}\left(t\right)\right)\right\}.$$ (14)

The updating scheme follows: $J_{i,j}^{n+1}$ = $J_{i,j}^{n}$ + *γ*$\frac{\partial \;\ell }{\partial \;J_{i,j}}$|_*n*_. Here, *n* is the iteration number and *γ* is the learning rate.

### Monte Carlo Simulations for the Ising Model

All scripts were developed and executed in MATLAB R2018a on a Windows 10 machine with Intel i7 CPU@ 2.8 GHz and 16 GB of RAM. We used a Markov chain Monte Carlo (MCMC) method based on the metropolis algorithm to calculate the observables of the Ising model using the networks inferred from the pMEM and FSE. Here we present the simulations performed step by step:

(1)  Define the parameters **J** (network inferred with pMEM or FSE), the number of runs **t**, and a range of *β*_*simulated*_.
(2)  For each run randomly fix an ***s***_*i*_ from the configuration and compute the Hamiltonian *H*(***s***_*i*_).
(3)  If *H*(***s***_*i*_) ≤ 0 or *rand*(0, 1) ≤ exp$\left(\frac{H\left(s_{i}\right)}{\beta_{\text{simulated}}}\right)$, flip the state. Note: The command *rand*(0, 1) generates a random value between 0 and 1. Complete this for all elements in the configuration.
(4)  The final configuration of states is then used as the input for the next run.
(5)  Concatenate all runs into an *N* × *t* array and compute the averages of the observables (i.e., Pearson correlation <*s*_*i*_***s***_*j*_>, magnetization |**M**|, susceptibility **χ**).
(6)  Do this for all *β*_*simulated*_.

Because of the computational cost, when performing MCMC simulations for the grid-search parameter optimization we used *t* = 2,000 runs and *β*_*simulated*_ from 0.2 to 3.0 with increments of 0.2. For the control case based on pMEM, we used *t* = *N* × *N* × 10 runs with *β*_*simulated*_ from 1 to 20 with increments of 0.5. Last, when evaluating the thermodynamic properties magnetization |**M**|, susceptibility **χ** using the rs-SC network, we use *t* = 100,000 with *β*_*simulated*_ from 0.2 to 3.0 with increments of 0.05. The number of runs as well as range and increments of *β*_*simulated*_ were selected based on the task performed to maximize algorithmic performance and to minimize processing time. The upper and lower bound of these values was first empirically determined to be containing the optimal range by simulations.

### Phase Transition and Biological Motivation

The simplicity of the Ising model enables the prediction of cooperative behavior among a system of biological elements wherein each element has two states, and the energy of the system depends only on the state of each element and its neighbors. Moreover, the model parameters and representative physical properties are readily amenable to biological interpretation in the context of various complex systems. For example, a four-dimensional cellular automaton-like Ising model has been previously developed to investigate transitions between normal, proliferative, hypoxic, and necrotic states in the tumorigenesis processes (Durrett, 2013; Torquato, 2011). Ising-like models have also been implemented to estimate information transfer between spins occurring on the human connectome (Marinazzo et al., 2014) or to assess differentially expressed genes in cancer patients (X. Li et al., 2011), and even to model the joint expression profiles of genes to reconstruct E. coli gene interaction pathways (Santhanam et al., 2009). Hence, when we discuss a “phase transition,” it is a result of the interactions among many elements, not from the specific nature of the individual units (be they ferromagnetic materials or biological elements like neurons, protein chains, or genes).

To evaluate these transitions, we look to the average of activations over the whole network (termed magnetization), which determines the ordering of the system. Magnetic susceptibility is simply the variance of the magnetization. If all the binary spin states are aligned in the same direction, a magnetization of ±1 corresponds to a configuration of complete order. The magnetization per site, defined as *M* = $\sum_{i=1}^{N}$ <***s***_*i*_>, where < · > represents the ensemble average and quantifies the mean tendency that ***s***_*i*_ = 1 as opposed to ***s***_*i*_ = −1, is taken across the brain regions. The magnetic susceptibility is defined as *χ* = $\frac{1}{\beta }$ (<*M*^2^> − <*M*>^2^) (Landau, 2009).

Here, we consider brain networks positioned near a critical point between complete inactivity (i.e., neuronal death) and random activity (as in epilepsy, for example). In a less extreme sense, simulations of Ising dynamics can reveal a transition from a hypoactive state towards a more chaotic state. As described in Equation 9, the behavior of the modeled system depends on temperature. However, for a network of neurons or brain regions, there is no real concept of “temperature.” Hence, when performing Monte Carlo simulations of the Ising model, we may describe temperature (*T*) as a “tolerance” of the system in the sense that the effect of the *T* parameter injects additional randomness into the simulated dynamics of the system. Thus, for very low *T* (*T* < *T*_*critical*_), spontaneous MCMC spin flips are less probable, with the spins in each configuration mostly aligned to contribute the minimum energy of the system. For very high *T* (*T* > *T*_*critical*_), the magnetic ordering is completely lost as a result of a high number of spontaneous spin flips; thus, the magnetization tends to zero, which can be used to characterize the disordered (or chaotic) phase. In the intermediate range of *T* where self-organized criticality and second-order phase transitions occur, there is a point of maximal fluctuations in the magnetization at *T* = *T*_*critical*_ that corresponds to a peak in the magnetic susceptibility (Chialvo, 2010). Thus, a system with lower critical temperature is suggestive of a lower tolerance to perturbations in the network as determined via Monte Carlo simulations of brain dynamics than is a higher critical temperature that would suggest a higher tolerance.

### Parameter Optimization Using a Similarity Metric and Correlation Function

In this work, we use a grid-search optimization scheme to find the optimal parameters {*β*, *A*}. The parameters are evaluated from 0.2 to 3.0 with 0.2 increments for all 76 participants. With the FSE, **J**, we generate a correlation function *max* *f*_*c*_(*β*, *A*) by simulating the Ising model with Monte Carlo simulations, computing a Pearson correlation between observed and simulated functional connectivity for all *β*_*simulated*_ (from 0.2 to 3.0 with 0.2 increments). Further, we compute a similarity metric *S*_*m*_(*β*, *A*) via the correlation *r*(|*J*_*i*,*j*_|, *W*_*i*,*j*_) ∀ *i*, *j* to ensure that |*J*_*i*,*j*_| ∝ *W*_*i*,*j*_, the structural connectome. To identify the optimal parameters, we find *A*, *β* such that *f*(*β*, *A*) = *max* *f*_*c*_ + *S*_*m*_ is maximized.

### Excitation-Inhibition (E/I) Ratio

It is important to note that in using the terminology connectome-level excitation-inhibition balance and hyperexcitation, we are not necessarily inferring directionality of these interactions or measuring processes at a neuronal level. Rather, we used such terminology to bridge the gap between microscale interactions (such as excitation and inhibition of neuronal circuits) and the connectome-level changes that may occur because of such processes. Note that similar terminologies have previously been adopted in several seminal studies that investigated neuronal firing patterns using the Ising model (Schneidman et al., 2006; Tkačik et al., 2013). To be clear, from a connectomics perspective, if several brain regions are identified to have an increase in positive edges in the rs-SC, collectively, that would suggest a wider spread pattern of coupling (i.e., more likely to exhibit a pattern of global coupling) that may subserve hyperexcitation. It is in this context that we conceptualize the excitation-inhibition (E/I) ratio, a global (whole-brain) or local (ROI-level) estimation of E/I balance, computed as the sum of positive edges divided by the sum of negative edges. For example, if an ROI in the network has 45 positive edges and 34 negative edges, then the E/I ratio = $\frac{45}{34}$, or 1.32 (a value of 1 indicates perfect E/I balance).

## SUPPORTING INFORMATION

Supporting information for this article is available at https://doi.org/10.1162/netn_a_00220.

## AUTHOR CONTRIBUTIONS

Igor Fortel: Conceptualization; Formal analysis; Investigation; Methodology; Software; Validation; Visualization; Writing – original draft; Writing – review & editing. Mitchell Butler: Conceptualization; Methodology; Writing – review & editing. Laura E. Korthauer: Data curation; Investigation; Methodology. Liang Zhan: Conceptualization; Formal analysis; Funding acquisition; Methodology. Olusola Ajilore: Conceptualization; Investigation. Anastasios Sidiropoulos: Conceptualization; Investigation. Yichao Wu: Conceptualization; Investigation. Ira Driscoll: Data curation; Investigation. Dan Schonfeld: Conceptualization; Methodology; Supervision; Validation. Alex Leow: Conceptualization; Formal analysis; Funding acquisition; Methodology; Supervision; Validation; Writing – review & editing.

## FUNDING INFORMATION

Alex Leow and Liang Zhan, National Institutes of Health (https://dx.doi.org/10.13039/100000002), Award ID: R01AG071243. Alex Leow, Yichao Wu, and Liang Zhan, National Institutes of Health (https://dx.doi.org/10.13039/100000002), Award ID: R01MH125928. Liang Zhan, National Institutes of Health (https://dx.doi.org/10.13039/100000002), Award ID: U01AG068057. Liang Zhan, National Science Foundation (https://dx.doi.org/10.13039/100000001), Award ID: IIS2045848. Liang Zhan, National Science Foundation (https://dx.doi.org/10.13039/100000001), Award ID: IIS1837956.

## TECHNICAL TERMS

Criticality: From physics, representing the state of a dynamical system between order and disorder.
Functional connectivity (FC): Undirected measure describing the statistical dependance between brain regions based on blood oxygen level–dependent (BOLD) signals from fMRI.
Pairwise maximum entropy model (pMEM): A maximum entropy model that takes into account the average activity of a node as well as pairwise interactions.
Ising model: Network model in which node activity is represented with binary states and energy is computed based on pairwise interactions.
Maximum entropy model (MEM): A statistical model of data representing the highest entropy that satisfies the constraint of prior knowledge.
Structural connectome (SC): Network representation of physical connections in the brain. Nodes represent brain regions. Edges represent a measure of connectivity between them.
APOE-ε4: An allele representing one of the strongest genetic risk factors for developing Alzheimer’s disease (AD).
Hamiltonian: Equation representing the total energy of the system.
Boltzmann distribution: Quantifies the probability that a system will be in a specific state based on energy (of that state) and temperature (system).
Critical temperature: Parameter describing the tolerance of a system to increasing perturbations (commonly used in physics to identify loss of magnetic properties).

## References

[bib1] Aboud, O., Mrak, R. E., Boop, F. A., & Griffin, W. S. T. (2013). Epilepsy: Neuroinflammation, neurodegeneration, and APOE genotype. Acta Neuropathologica Communications, 1(1), 41. 10.1186/2051-5960-1-41, 24252240 PMC3893449

[bib2] Aksenov, D. P., Li, L., Miller, M. J., & Wyrwicz, A. M. (2019). Role of the inhibitory system in shaping the BOLD fMRI response. NeuroImage, 201, 116034. 10.1016/j.neuroimage.2019.116034, 31326573 PMC6886666

[bib3] Alexander-Bloch, A. F., Vértes, P. E., Stidd, R., Lalonde, F., Clasen, L., Rapoport, J., Giedd, J., Bullmore, E. T., & Gogtay, N. (2013). The anatomical distance of functional connections predicts brain network topology in health and schizophrenia. Cerebral Cortex, 23(1), 127–138. 10.1093/cercor/bhr388, 22275481 PMC3513955

[bib4] Andersson, J. L. R., & Sotiropoulos, S. N. (2016). An integrated approach to correction for off-resonance effects and subject movement in diffusion MR imaging. NeuroImage, 125, 1063–1078. 10.1016/j.neuroimage.2015.10.019, 26481672 PMC4692656

[bib5] Ashourvan, A., Gu, S., Mattar, M. G., Vettel, J. M., & Bassett, D. S. (2017). The energy landscape underpinning module dynamics in the human brain connectome. NeuroImage, 157, 364–380. 10.1016/j.neuroimage.2017.05.067, 28602945 PMC5600845

[bib6] Ashourvan, A., Shah, P., Pines, A., Gu, S., Lynn, C. W., Bassett, D. S., Davis, K. A., & Litt, B. (2021). Pairwise maximum entropy model explains the role of white matter structure in shaping emergent activation states. Communications Biology, 4(1), 1–15. 10.1038/s42003-021-01700-6, 33594239 PMC7887247

[bib7] Barrick, T. R., Charlton, R. A., Clark, C. A., & Markus, H. S. (2010). White matter structural decline in normal ageing: A prospective longitudinal study using tract-based spatial statistics. NeuroImage, 51(2), 565–577. 10.1016/j.neuroimage.2010.02.033, 20178850

[bib8] Beggs, J. M., & Plenz, D. (2003). Neuronal avalanches in neocortical circuits. Journal of Neuroscience, 23(35), 11167–11177. 10.1523/JNEUROSCI.23-35-11167.2003, 14657176 PMC6741045

[bib9] Behrens, T. E. J., Berg, H. J., Jbabdi, S., Rushworth, M. F. S., & Woolrich, M. W. (2007). Probabilistic diffusion tractography with multiple fibre orientations: What can we gain? NeuroImage, 34(1), 144–155. 10.1016/j.neuroimage.2006.09.018, 17070705 PMC7116582

[bib10] Behrens, T. E. J., Woolrich, M. W., Jenkinson, M., Johansen-Berg, H., Nunes, R. G., Clare, S., Matthews, P. M., Brady, J. M., & Smith, S. M. (2003). Characterization and propagation of uncertainty in diffusion-weighted MR imaging. Magnetic Resonance in Medicine, 50(5), 1077–1088. 10.1002/mrm.10609, 14587019

[bib11] Besag, J. (1975). Statistical analysis of non-lattice data. Journal of the Royal Statistical Society: Series D (The Statistician), 24(3), 179–195. 10.2307/2987782

[bib12] Besag, J. (1977). Efficiency of pseudolikelihood estimation for simple Gaussian fields. Biometrika, 64(3), 616–618. 10.2307/2345341

[bib13] Bettinardi, R. G., Deco, G., Karlaftis, V. M., Van Hartevelt, T. J., Fernandes, H. M., Kourtzi, Z., Kringelbach, M. L., & Zamora-López, G. (2017). How structure sculpts function: Unveiling the contribution of anatomical connectivity to the brain’s spontaneous correlation structure. Chaos, 27(4), 047409. 10.1063/1.4980099, 28456160

[bib14] Bi, D., Wen, L., Wu, Z., & Shen, Y. (2020). GABAergic dysfunction in excitatory and inhibitory (E/I) imbalance drives the pathogenesis of Alzheimer’s disease. Alzheimer’s and Dementia, 16(9), 1312–1329. 10.1002/alz.12088, 32543726

[bib15] Bretsky, P. M., Buckwalter, J. G., Seeman, T. E., Miller, C. A., Poirier, J., Schellenberg, G. D., Finch, C. E., & Henderson, V. W. (1999). Evidence for an interaction between apolipoprotein E genotype, gender, and Alzheimer disease. Alzheimer Disease and Associated Disorders, 13(4), 216–221. 10.1097/00002093-199910000-00007, 10609670

[bib16] Bruining, H., Hardstone, R., Juarez-Martinez, E. L., Sprengers, J., Avramiea, A.-E., Simpraga, S., Houtman, S. J., Poil, S.-S., Dallares, E., Palva, S., Oranje, B., Matias Palva, J., Mansvelder, H. D., & Linkenkaer-Hansen, K. (2020). Measurement of excitation-inhibition ratio in autism spectrum disorder using critical brain dynamics. Scientific Reports, 10(1), 9195. 10.1038/s41598-020-65500-4, 32513931 PMC7280527

[bib17] Busche, M. A., & Konnerth, A. (2016). Impairments of neural circuit function in Alzheimer’s disease. Philosophical Transactions of the Royal Society B: Biological Sciences, 371(1700). 10.1098/rstb.2015.0429, 27377723 PMC4938029

[bib18] Chételat, G., Landeau, B., Salmon, E., Yakushev, I., Bahri, M. A., Mézenge, F., Perrotin, A., Bastin, C., Manrique, A., Scheurich, A., Scheckenberger, M., Desgranges, B., Eustache, F., & Fellgiebel, A. (2013). Relationships between brain metabolism decrease in normal aging and changes in structural and functional connectivity. NeuroImage, 76, 167–177. 10.1016/j.neuroimage.2013.03.009, 23518010

[bib19] Chialvo, D. R. (2010). Emergent complex neural dynamics. Nature Physics, 6(10), 744–750. 10.1038/nphys1803

[bib20] Cocchi, L., Gollo, L. L., Zalesky, A., & Breakspear, M. (2017). Criticality in the brain: A synthesis of neurobiology, models and cognition. Progress in Neurobiology, 158, 132–152. 10.1016/j.pneurobio.2017.07.002, 28734836

[bib21] Cocco, S., Monasson, R., Posani, L., & Tavoni, G. (2017). Functional networks from inverse modeling of neural population activity. Current Opinion in Systems Biology, 3, 103–110. 10.1016/j.coisb.2017.04.017

[bib22] Cofré, R., Herzog, R., Corcoran, D., & Rosas, F. E. (2019). A comparison of the maximum entropy principle across biological spatial scales. Entropy, 21(10), 1009. 10.3390/e21101009

[bib23] Cox, R. W. (1996). AFNI: Software for analysis and visualization of functional magnetic resonance neuroimages. Computers and Biomedical Research, 29(3), 162–173. 10.1006/cbmr.1996.0014, 8812068

[bib24] Curiati, P. K., Tamashiro-Duran, J. H., Duran, F. L. S., Buchpiguel, C. A., Squarzoni, P., Romano, D. C., Vallada, H., Menezes, P. R., Scazufca, M., Busatto, G. F., & Alves, T. C. T. F. (2011). Age-related metabolic profiles in cognitively healthy elders: Results from a voxel-based [18F]fluorodeoxyglucose-positron-emission tomography study with partial volume effects correction. American Journal of Neuroradiology, 32(3), 560–565. 10.3174/ajnr.A2321, 21273352 PMC8013092

[bib25] Dale, A. M., Fischl, B., & Sereno, M. I. (1999). Cortical surface-based analysis: I. Segmentation and surface reconstruction. NeuroImage, 9(2), 179–194. 10.1006/nimg.1998.0395, 9931268

[bib26] Damoiseaux, J. S., Beckmann, C. F., Arigita, E. J. S., Barkhof, F., Scheltens, P., Stam, C. J., Smith, S. M., & Rombouts, S. A. R. B. (2008). Reduced resting-state brain activity in the “default network” in normal aging. Cerebral Cortex, 18(8), 1856–1864. 10.1093/cercor/bhm207, 18063564

[bib27] Deco, G., Jirsa, V. K., Robinson, P. A., Breakspear, M., & Friston, K. (2008). The dynamic brain: From spiking neurons to neural masses and cortical fields. PLoS Computational Biology, 4(8), e1000092. 10.1371/journal.pcbi.1000092, 18769680 PMC2519166

[bib28] DeTure, M. A., & Dickson, D. W. (2019). The neuropathological diagnosis of Alzheimer’s disease. Molecular Neurodegeneration, 14(1), 32. 10.1186/s13024-019-0333-5, 31375134 PMC6679484

[bib29] Durrett, R. (2013). Cancer modeling: A personal perspective. Notices of the AMS, 60(3), 304–309. 10.1090/noti953

[bib30] Ezaki, T., Fonseca dos Reis, E., Watanabe, T., Sakaki, M., & Masuda, N. (2020). Closer to critical resting-state neural dynamics in individuals with higher fluid intelligence. Communications Biology, 3(1), 1–9. 10.1038/s42003-020-0774-y, 32015402 PMC6997374

[bib31] Ezaki, T., Watanabe, T., Ohzeki, M., & Masuda, N. (2017). Energy landscape analysis of neuroimaging data. Philosophical Transactions of the Royal Society A: Mathematical, Physical and Engineering Sciences, 375(2096), 20160287. 10.1098/rsta.2016.0287, 28507232 PMC5434078

[bib32] Fischl, B., Salat, D. H., Busa, E., Albert, M., Dieterich, M., Haselgrove, C., Kouwe, A. van der, Killiany, R., Kennedy, D., Klaveness, S., Montillo, A., Makris, N., Rosen, B., & Dale, A. M. (2002). Whole brain segmentation: Automated labeling of neuroanatomical structures in the human brain. Neuron, 33(3), 341–355. 10.1016/S0896-6273(02)00569-X, 11832223

[bib33] Fischl, B., van der Kouwe, A., Destrieux, C., Halgren, E., Ségonne, F., Salat, D. H., Busa, E., Seidman, L. J., Goldstein, J., Kennedy, D., Caviness, V., Makris, N., Rosen, B., & Dale, A. M. (2004). Automatically parcellating the human cerebral cortex. Cerebral Cortex, 14(1), 11–22. 10.1093/cercor/bhg087, 14654453

[bib34] Folstein, M. F., Folstein, S. E., & McHugh, P. R. (1975). “Mini–mental state”: A practical method for grading the cognitive state of patients for the clinician. Journal of Psychiatric Research, 12(3), 189–198. 10.1016/0022-3956(75)90026-6 1202204

[bib35] Fortel, I., Butler, M., Korthauer, L. E., Zhan, L., Ajilore, O., Driscoll, I., Sidiropoulos, A., Zhang, Y., Guo, L., Huang, H., Schonfeld, D., & Leow, A. (2019). Brain dynamics through the lens of statistical mechanics by unifying structure and function. In D. Shen, T. Liu, T. M. Peters, L. H. Staib, C. Essert, S. Zhou, P.-T. Yap, & A. Khan (Eds.), Medical image computing and computer assisted intervention – MICCAI 2019 (pp. 503–511). Springer International Publishing. 10.1007/978-3-030-32254-0_56

[bib36] Fortel, I., Korthauer, L. E., Morrissey, Z., Zhan, L., Ajilore, O., Wolfson, O., Driscoll, I., Schonfeld, D., & Leow, A. (2020). Connectome signatures of hyperexcitation in cognitively intact middle-aged female APOE-ε4 carriers. Cerebral Cortex, 30(12), 6350–6362. 10.1093/cercor/bhaa190, 32662517 PMC7609923

[bib37] Gu, S., Cieslak, M., Baird, B., Muldoon, S. F., Grafton, S. T., Pasqualetti, F., & Bassett, D. S. (2018). The energy landscape of neurophysiological activity implicit in brain network structure. Scientific Reports, 8(1), 2507. 10.1038/s41598-018-20123-8, 29410486 PMC5802783

[bib38] Hahn, G., Ponce-Alvarez, A., Monier, C., Benvenuti, G., Kumar, A., Chavane, F., Deco, G., & Frégnac, Y. (2017). Spontaneous cortical activity is transiently poised close to criticality. PLoS Computational Biology, 13(5), e1005543. 10.1371/journal.pcbi.1005543, 28542191 PMC5464673

[bib39] Haimovici, A., Tagliazucchi, E., Balenzuela, P., & Chialvo, D. R. (2013). Brain organization into resting state networks emerges at criticality on a model of the human connectome. Physical Review Letters, 110(17), 178101. 10.1103/PhysRevLett.110.178101, 23679783

[bib40] Heiney, K., Ramstad, O. H., Sandvig, I., Sandvig, A., & Nichele, S. (2019). Assessment and manipulation of the computational capacity of in vitro neuronal networks through criticality in neuronal avalanches. 2019 IEEE Symposium Series on Computational Intelligence (SSCI), 247–254. 10.1109/SSCI44817.2019.9002693

[bib41] Hijazi, S., Heistek, T., Scheltens, P., Mansvelder, H. D., Smit, A. B., & van Kesteren, R. E. (2020). Interneuron hyperexcitability as both causal factor and risk factor in Alzheimer’s disease. Alzheimer’s and Dementia, 16(S3), e040877. 10.1002/alz.040877

[bib42] Honey, C. J., Sporns, O., Cammoun, L., Gigandet, X., Thiran, J. P., Meuli, R., & Hagmann, P. (2009). Predicting human resting-state functional connectivity from structural connectivity. Proceedings of the National Academy of Sciences, 106(6), 2035–2040. 10.1073/pnas.0811168106, 19188601 PMC2634800

[bib43] Jack, C. R., Wiste, H. J., Weigand, S. D., Knopman, D. S., Vemuri, P., Mielke, M. M., Lowe, V., Senjem, M. L., Gunter, J. L., Machulda, M. M., Gregg, B. E., Pankratz, V. S., Rocca, W. A., & Petersen, R. C. (2015). Age, sex, and APOE ε4 effects on memory, brain structure, and β-amyloid across the adult life span. JAMA Neurology, 72(5), 511–519. 10.1001/jamaneurol.2014.4821, 25775353 PMC4428984

[bib44] Jenkinson, M., Bannister, P., Brady, M., & Smith, S. (2002). Improved optimization for the robust and accurate linear registration and motion correction of brain images. NeuroImage, 17(2), 825–841. 10.1006/nimg.2002.1132, 12377157

[bib45] Jiménez-Balado, J., & Eich, T. S. (2021). GABAergic dysfunction, neural network hyperactivity and memory impairments in human aging and Alzheimer’s disease. Seminars in Cell and Developmental Biology, 116, 146–159. 10.1016/j.semcdb.2021.01.005, 33573856 PMC8292162

[bib46] Johnson-Greene, D. (2004). Dementia Rating Scale-2 (DRS-2). By P. J. Jurica, C. L. Leitten, and S. Mattis: Psychological assessment resources, 2001. Archives of Clinical Neuropsychology, 19, 145–147. 10.1016/j.acn.2003.07.003

[bib47] Kadirvelu, B., Hayashi, Y., & Nasuto, S. J. (2017). Inferring structural connectivity using Ising couplings in models of neuronal networks. Scientific Reports, 7(1), 1–12. 10.1038/s41598-017-05462-2, 28811468 PMC5557813

[bib48] Kinouchi, O., & Copelli, M. (2006). Optimal dynamical range of excitable networks at criticality. Nature Physics, 2(5), 348–351. 10.1038/nphys289

[bib49] Koelewijn, L., Lancaster, T. M., Linden, D., Dima, D. C., Routley, B. C., Magazzini, L., Barawi, K., Brindley, L., Adams, R., Tansey, K. E., Bompas, A., Tales, A., Bayer, A., & Singh, K. (2019). Oscillatory hyperactivity and hyperconnectivity in young APOE-ɛ4 carriers and hypoconnectivity in Alzheimer’s disease. eLife, 8, e36011. 10.7554/eLife.36011, 31038453 PMC6491037

[bib50] Korthauer, L. E., Zhan, L., Ajilore, O., Leow, A., & Driscoll, I. (2018). Disrupted topology of the resting state structural connectome in middle-aged APOE ε4 carriers. NeuroImage, 178, 295–305. 10.1016/j.neuroimage.2018.05.052, 29803958 PMC6249680

[bib51] Koutsodendris, N., Nelson, M. R., Rao, A., & Huang, Y. (2022). Apolipoprotein E and Alzheimer’s disease: Findings, hypotheses, and potential mechanisms. Annual Review of Pathology: Mechanisms of Disease, 17(1). 10.1146/annurev-pathmechdis-030421-112756, 34460318

[bib52] Krishnan, G. P., González, O. C., & Bazhenov, M. (2018). Origin of slow spontaneous resting-state neuronal fluctuations in brain networks. Proceedings of the National Academy of Sciences, 115(26), 6858–6863. 10.1073/pnas.1715841115, 29884650 PMC6042137

[bib53] Landau, D. P. (2009). A guide to Monte Carlo simulations in statistical physics. Retrieved from https://www.amazon.com/Guide-Monte-Simulations-Statistical-Physics/dp/0521768489. 10.1017/CBO9780511994944

[bib54] Leung, L., Andrews-Zwilling, Y., Yoon, S. Y., Jain, S., Ring, K., Dai, J., Wang, M. M., Tong, L., Walker, D., & Huang, Y. (2012). Apolipoprotein E4 causes age- and sex-dependent impairments of hilar GABAergic interneurons and learning and memory deficits in mice. PLoS ONE, 7(12), e53569. 10.1371/journal.pone.0053569, 23300939 PMC3534053

[bib55] Li, X., Feltus, F. A., Sun, X., Wang, J. Z., & Luo, F. (2011). Identifying differentially expressed genes in cancer patients using a non-parameter Ising model. Proteomics, 11(19), 3845–3852. 10.1002/pmic.201100180, 21761563

[bib56] Li, Y., Sun, H., Chen, Z., Xu, H., Bu, G., & Zheng, H. (2016). Implications of GABAergic neurotransmission in Alzheimer’s disease. Frontiers in Aging Neuroscience, 8, 31. 10.3389/fnagi.2016.00031, 26941642 PMC4763334

[bib57] Lombardi, F., Herrmann, H. J., & de Arcangelis, L. (2017). Balance of excitation and inhibition determines 1/f power spectrum in neuronal networks. Chaos, 27(4), 047402. 10.1063/1.4979043, 28456161

[bib58] Marinazzo, D., Pellicoro, M., Wu, G., Angelini, L., Cortés, J. M., & Stramaglia, S. (2014). Information transfer and criticality in the Ising model on the human connectome. PLoS ONE, 9(4), e93616. 10.1371/journal.pone.0093616, 24705627 PMC3976308

[bib59] McDonald, C. R., McEvoy, L. K., Gharapetian, L., Fennema-Notestine, C., Hagler, D. J., Holland, D., Koyama, A., Brewer, J. B., Dale, A. M., & Alzheimer’s Disease Neuroimaging Initiative. (2009). Regional rates of neocortical atrophy from normal aging to early Alzheimer disease. Neurology, 73(6), 457–465. 10.1212/WNL.0b013e3181b16431, 19667321 PMC2727145

[bib60] Michielse, S., Coupland, N., Camicioli, R., Carter, R., Seres, P., Sabino, J., & Malykhin, N. (2010). Selective effects of aging on brain white matter microstructure: A diffusion tensor imaging tractography study. NeuroImage, 52(4), 1190–1201. 10.1016/j.neuroimage.2010.05.019, 20483378

[bib61] Montez, T., Poil, S.-S., Jones, B. F., Manshanden, I., Verbunt, J. P. A., van Dijk, B. W., Brussaard, A. B., van Ooyen, A., Stam, C. J., Scheltens, P., & Linkenkaer-Hansen, K. (2009). Altered temporal correlations in parietal alpha and prefrontal theta oscillations in early-stage Alzheimer disease. Proceedings of the National Academy of Sciences, 106(5), 1614–1619. 10.1073/pnas.0811699106, 19164579 PMC2635782

[bib62] Najm, R., Jones, E. A., & Huang, Y. (2019). Apolipoprotein E4, inhibitory network dysfunction, and Alzheimer’s disease. Molecular Neurodegeneration, 14(1), 24. 10.1186/s13024-019-0324-6, 31186040 PMC6558779

[bib63] Nghiem, T.-A., Telenczuk, B., Marre, O., Destexhe, A., & Ferrari, U. (2018). Maximum-entropy models reveal the excitatory and inhibitory correlation structures in cortical neuronal activity. Physical Review E, 98(1), 012402. 10.1103/PhysRevE.98.012402, 30110850

[bib64] Nguyen, H. C., Zecchina, R., & Berg, J. (2017). Inverse statistical problems: From the inverse Ising problem to data science. Advances in Physics, 66(3), 197–261. 10.1080/00018732.2017.1341604

[bib65] Niu, W., Huang, X., Xu, K., Jiang, T., & Yu, S. (2019). Pairwise interactions among brain regions organize large-scale functional connectivity during execution of various tasks. Neuroscience, 412, 190–206. 10.1016/j.neuroscience.2019.05.011, 31181368

[bib66] Nuriel, T., Angulo, S. L., Khan, U., Ashok, A., Chen, Q., Figueroa, H. Y., Emrani, S., Liu, L., Herman, M., Barrett, G., Savage, V., Buitrago, L., Cepeda-Prado, E., Fung, C., Goldberg, E., Gross, S. S., Hussaini, S. A., Moreno, H., Small, S. A., & Duff, K. E. (2017). Neuronal hyperactivity due to loss of inhibitory tone in APOE4 mice lacking Alzheimer’s disease-like pathology. Nature Communications, 8(1), 1464. 10.1038/s41467-017-01444-0, 29133888 PMC5684208

[bib67] Nuzzi, D., Pellicoro, M., Angelini, L., Marinazzo, D., & Stramaglia, S. (2020). Synergistic information in a dynamical model implemented on the human structural connectome reveals spatially distinct associations with age. Network Neuroscience, 4(3), 910–924. 10.1162/netn_a_00146, 33615096 PMC7888489

[bib68] Ostojic, S., & Brunel, N. (2011). From spiking neuron models to linear-nonlinear models. PLoS Computational Biology, 7(1), e1001056. 10.1371/journal.pcbi.1001056, 21283777 PMC3024256

[bib69] Palop, J. J., Chin, J., Roberson, E. D., Wang, J., Thwin, M. T., Bien-Ly, N., Yoo, J., Ho, K. O., Yu, G.-Q., Kreitzer, A., Finkbeiner, S., Noebels, J. L., & Mucke, L. (2007). Aberrant excitatory neuronal activity and compensatory remodeling of inhibitory hippocampal circuits in mouse models of Alzheimer’s disease. Neuron, 55(5), 697–711. 10.1016/j.neuron.2007.07.025, 17785178 PMC8055171

[bib70] Paterno, R., Casalia, M., & Baraban, S. C. (2020). Interneuron deficits in neurodevelopmental disorders: Implications for disease pathology and interneuron-based therapies. European Journal of Paediatric Neurology, 24, 81–88. 10.1016/j.ejpn.2019.12.015, 31870698 PMC7152321

[bib71] Payami, H., Montee, K. R., Kaye, J. A., Bird, T. D., Yu, C. E., Wijsman, E. M., & Schellenberg, G. D. (1994). Alzheimer’s disease, apolipoprotein E4, and gender. JAMA, 271(17), 1316–1317. 10.1001/jama.1994.03510410028015, 8158809

[bib72] Petrache, A. L., Rajulawalla, A., Shi, A., Wetzel, A., Saito, T., Saido, T. C., Harvey, K., & Ali, A. B. (2019). Aberrant excitatory–inhibitory synaptic mechanisms in entorhinal cortex microcircuits during the pathogenesis of Alzheimer’s disease. Cerebral Cortex, 29(4), 1834–1850. 10.1093/cercor/bhz016, 30766992 PMC6418384

[bib73] Rabuffo, G., Fousek, J., Bernard, C., & Jirsa, V. (2021). Neuronal cascades shape whole-brain functional dynamics at rest. eNeuro, 8(5). 10.1523/ENEURO.0283-21.2021, 34583933 PMC8555887

[bib74] Rajkumar, R., Régio Brambilla, C., Veselinović, T., Bierbrier, J., Wyss, C., Ramkiran, S., Orth, L., Lang, M., Rota Kops, E., Mauler, J., Scheins, J., Neumaier, B., Ermert, J., Herzog, H., Langen, K.-J., Binkofski, F. C., Lerche, C., Shah, N. J., & Neuner, I. (2021). Excitatory–inhibitory balance within EEG microstates and resting-state fMRI networks: Assessed via simultaneous trimodal PET–MR–EEG imaging. Translational Psychiatry, 11(1), 1–15. 10.1038/s41398-020-01160-2, 33462192 PMC7813876

[bib75] Reichl, L. E., & Luscombe, J. H. (1999). *A modern course in statistical physics*, 2nd edition [Book review]. American Journal of Physics, 67(12), 1285–1287. 10.1119/1.19118

[bib76] Ren, S.-Q., Yao, W., Yan, J.-Z., Jin, C., Yin, J.-J., Yuan, J., Yu, S., & Cheng, Z. (2018). Amyloid β causes excitation/inhibition imbalance through dopamine receptor 1-dependent disruption of fast-spiking GABAergic input in anterior cingulate cortex. Scientific Reports, 8. 10.1038/s41598-017-18729-5, 29321592 PMC5762926

[bib77] Rodrigue, K. M., Kennedy, K. M., Devous, M. D., Rieck, J. R., Hebrank, A. C., Diaz-Arrastia, R., Mathews, D., & Park, D. C. (2012). β-Amyloid burden in healthy aging: Regional distribution and cognitive consequences. Neurology, 78(6), 387–395. 10.1212/WNL.0b013e318245d295, 22302550 PMC3280058

[bib78] Roudi, Y., Tyrcha, J., & Hertz, J. (2009). Ising model for neural data: Model quality and approximate methods for extracting functional connectivity. Physical Review E, 79(5), 051915. 10.1103/PhysRevE.79.051915, 19518488

[bib79] Rowe, C. C., Ellis, K. A., Rimajova, M., Bourgeat, P., Pike, K. E., Jones, G., Fripp, J., Tochon-Danguy, H., Morandeau, L., O’Keefe, G., Price, R., Raniga, P., Robins, P., Acosta, O., Lenzo, N., Szoeke, C., Salvado, O., Head, R., Martins, R., … Villemagne, V. L. (2010). Amyloid imaging results from the Australian Imaging, Biomarkers and Lifestyle (AIBL) study of aging. Neurobiology of Aging, 31(8), 1275–1283. 10.1016/j.neurobiolaging.2010.04.007, 20472326

[bib80] Santhanam, N., Dingel, J., & Milenkovic, O. (2009). On modeling gene regulatory networks using Markov random fields. In 2009 IEEE Information Theory Workshop on Networking and Information Theory (pp. 156–160). IEEE. 10.1109/ITWNIT.2009.5158562

[bib81] Schneidman, E., Berry, M. J., Segev, R., & Bialek, W. (2006). Weak pairwise correlations imply strongly correlated network states in a neural population. Nature, 440(7087), 1007–1012. 10.1038/nature04701, 16625187 PMC1785327

[bib82] Schuff, N., Amend, D. L., Knowlton, R., Norman, D., Fein, G., & Weiner, M. W. (1999). Age-related metabolite changes and volume loss in the hippocampus by magnetic resonance spectroscopy and imaging. Neurobiology of Aging, 20(3), 279–285. 10.1016/s0197-4580(99)00022-6, 10588575 PMC2733348

[bib83] Sheline, Y. I., Morris, J. C., Snyder, A. Z., Price, J. L., Yan, Z., D’Angelo, G., Liu, C., Dixit, S., Benzinger, T., Fagan, A., Goate, A., & Mintun, M. A. (2010). APOE4 allele disrupts resting state fMRI connectivity in the absence of amyloid plaques or decreased CSF Aβ42. Journal of Neuroscience, 30(50), 17035–17040. 10.1523/JNEUROSCI.3987-10.2010, 21159973 PMC3023180

[bib84] Shen, K., Hutchison, R. M., Bezgin, G., Everling, S., & McIntosh, A. R. (2015). Network structure shapes spontaneous functional connectivity dynamics. Journal of Neuroscience, 35(14), 5579–5588. 10.1523/JNEUROSCI.4903-14.2015, 25855174 PMC6605321

[bib85] Shew, W. L., Yang, H., Petermann, T., Roy, R., & Plenz, D. (2009). Neuronal avalanches imply maximum dynamic range in cortical networks at criticality. Journal of Neuroscience, 29(49), 15595–15600. 10.1523/JNEUROSCI.3864-09.2009, 20007483 PMC3862241

[bib86] Shew, W. L., Yang, H., Yu, S., Roy, R., & Plenz, D. (2011). Information capacity and transmission are maximized in balanced cortical networks with neuronal avalanches. Journal of Neuroscience, 31(1), 55–63. 10.1523/JNEUROSCI.4637-10.2011, 21209189 PMC3082868

[bib87] Shlens, J., Field, G. D., Gauthier, J. L., Grivich, M. I., Petrusca, D., Sher, A., Litke, A. M., & Chichilnisky, E. J. (2006). The structure of multi-neuron firing patterns in primate retina. Journal of Neuroscience, 26(32), 8254–8266. 10.1523/JNEUROSCI.1282-06.2006, 16899720 PMC6673811

[bib88] Smith, S. M. (2002). Fast robust automated brain extraction. Human Brain Mapping, 17(3), 143–155. 10.1002/hbm.10062, 12391568 PMC6871816

[bib89] Smith, S. M., Beckmann, C. F., Andersson, J., Auerbach, E. J., Bijsterbosch, J., Douaud, G., Duff, E., Feinberg, D. A., Griffanti, L., Harms, M. P., Kelly, M., Laumann, T., Miller, K. L., Moeller, S., Petersen, S., Power, J., Salimi-Khorshidi, G., Snyder, A. Z., Vu, A. T., … WU-Minn HCP Consortium. (2013). Resting-state fMRI in the Human Connectome Project. NeuroImage, 80, 144–168. 10.1016/j.neuroimage.2013.05.039, 23702415 PMC3720828

[bib90] Smith, S. M., Jenkinson, M., Woolrich, M. W., Beckmann, C. F., Behrens, T. E. J., Johansen-Berg, H., Bannister, P. R., De Luca, M., Drobnjak, I., Flitney, D. E., Niazy, R. K., Saunders, J., Vickers, J., Zhang, Y., De Stefano, N., Brady, J. M., & Matthews, P. M. (2004). Advances in functional and structural MR image analysis and implementation as FSL. NeuroImage, 23, S208–S219. 10.1016/j.neuroimage.2004.07.051, 15501092

[bib91] Sornette, D. (2006). Critical phenomena in natural sciences: Chaos, fractals, selforganization and disorder: Concepts and tools. Springer Science & Business Media.

[bib92] Sotero, R. C., & Trujillo-Barreto, N. J. (2007). Modelling the role of excitatory and inhibitory neuronal activity in the generation of the BOLD signal. NeuroImage, 35(1), 149–165. 10.1016/j.neuroimage.2006.10.027, 17234435

[bib93] Stam, C. J., Montez, T., Jones, B. F., Rombouts, S. A. R. B., van der Made, Y., Pijnenburg, Y. A. L., & Scheltens, P. (2005). Disturbed fluctuations of resting state EEG synchronization in Alzheimer’s disease. Clinical Neurophysiology, 116(3), 708–715. 10.1016/j.clinph.2004.09.022, 15721085

[bib94] Stargardt, A., Swaab, D. F., & Bossers, K. (2015). The storm before the quiet: Neuronal hyperactivity and Aβ in the presymptomatic stages of Alzheimer’s disease. Neurobiology of Aging, 36(1), 1–11. 10.1016/j.neurobiolaging.2014.08.014, 25444609

[bib95] Sten, S., Lundengård, K., Witt, S. T., Cedersund, G., Elinder, F., & Engström, M. (2017). Neural inhibition can explain negative BOLD responses: A mechanistic modelling and fMRI study. NeuroImage, 158, 219–231. 10.1016/j.neuroimage.2017.07.002, 28687518

[bib96] Tagliazucchi, E. (2017). The signatures of conscious access and its phenomenology are consistent with large-scale brain communication at criticality. Consciousness and Cognition, 55, 136–147. 10.1016/j.concog.2017.08.008, 28846872

[bib97] Tagliazucchi, E., Balenzuela, P., Fraiman, D., & Chialvo, D. R. (2012). Criticality in large-scale brain fMRI dynamics unveiled by a novel point process analysis. Frontiers in Physiology, 3. 10.3389/fphys.2012.00015, 22347863 PMC3274757

[bib98] Tkačik, G., Marre, O., Mora, T., Amodei, D., Berry, M. J., II, & Bialek, W. (2013). The simplest maximum entropy model for collective behavior in a neural network. Journal of Statistical Mechanics: Theory and Experiment, 2013(03), P03011. 10.1088/1742-5468/2013/03/P03011

[bib99] Tkačik, G., Mora, T., Marre, O., Amodei, D., Palmer, S. E., Berry, M. J., & Bialek, W. (2015). Thermodynamics and signatures of criticality in a network of neurons. Proceedings of the National Academy of Sciences, 112(37), 11508–11513. 10.1073/pnas.1514188112, 26330611 PMC4577210

[bib100] Tok, S., Ahnaou, A., & Drinkenburg, W. (2021). Functional neurophysiological biomarkers of early-stage Alzheimer’s disease: A perspective of network hyperexcitability in disease progression. Journal of Alzheimer’s Disease. 10.3233/JAD-210397, 34420957 PMC9484128

[bib101] Torquato, S. (2011). Toward an Ising model of cancer and beyond. Physical Biology, 8(1), 015017. 10.1088/1478-3975/8/1/015017, 21301063 PMC3151151

[bib102] Wang, L., Zang, Y., He, Y., Liang, M., Zhang, X., Tian, L., Wu, T., Jiang, T., & Li, K. (2006). Changes in hippocampal connectivity in the early stages of Alzheimer’s disease: Evidence from resting state fMRI. NeuroImage, 31(2), 496–504. 10.1016/j.neuroimage.2005.12.033, 16473024

[bib103] Watanabe, T., Hirose, S., Wada, H., Imai, Y., Machida, T., Shirouzu, I., Konishi, S., Miyashita, Y., & Masuda, N. (2013). A pairwise maximum entropy model accurately describes resting-state human brain networks. Nature Communications, 4, 1370. 10.1038/ncomms2388, 23340410 PMC3660654

[bib104] Wilting, J., & Priesemann, V. (2019). 25 years of criticality in neuroscience—Established results, open controversies, novel concepts. Current Opinion in Neurobiology, 58, 105–111. 10.1016/j.conb.2019.08.002, 31546053

[bib105] Yeh, F.-C., Tang, A., Hobbs, J. P., Hottowy, P., Dabrowski, W., Sher, A., Litke, A., & Beggs, J. M. (2010). Maximum entropy approaches to living neural networks. Entropy, 12(1), 89–106. 10.3390/e12010089

[bib106] Yesavage, J. A., Brink, T. L., Rose, T. L., Lum, O., Huang, V., Adey, M., & Leirer, V. O. (1982). Development and validation of a geriatric depression screening scale: A preliminary report. Journal of Psychiatric Research, 17(1), 37–49. 10.1016/0022-3956(82)90033-4, 7183759

[bib107] Zanoci, C., Dehghani, N., & Tegmark, M. (2019). Ensemble inhibition and excitation in the human cortex: An Ising-model analysis with uncertainties. Physical Review E, 99(3), 032408. 10.1103/PhysRevE.99.032408, 30999501

