# Supplementary material for: Inferring excitation-inhibition dynamics using a maximum entropy model unifying brain structure and function
Source: Netw Neurosci. 2022 Jun 1;6(2):420–44. doi: 10.1162/netn_a_00220 (PMC9205431; doi:10.1162/netn_a_00220)
Supplement: Supplementary file 1 [file netn-06-420-s001.pdf]

Supplementary Material

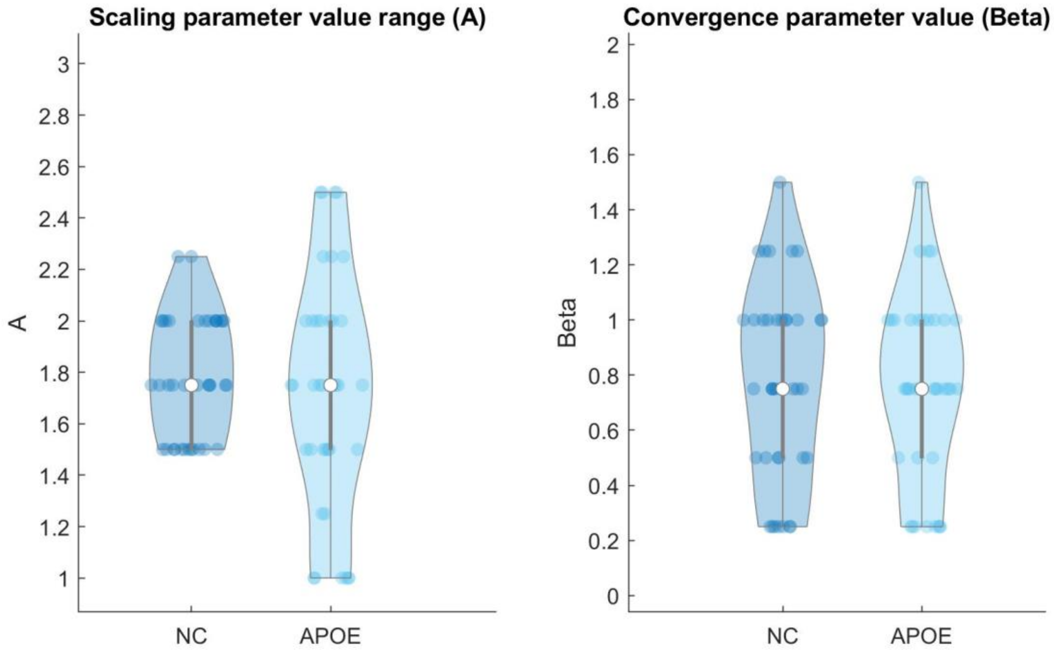

Supplementary Figure 1. Optimal parameter range for non-carrier and APOE - carrier groups

Presented here are the range of A and β, for both groups with A ranging from 1.5 to 2.25 in the NC group, and 1 to 2.5 in the APOE group. Both groups have a median of 1.75 for this parameter. Further, the β convergence parameter is more consistent between groups ranging from 0.25 to 1.5 for both, with a median of 0.75 for both groups. We note here that the parameters tested were 0.25 to 3.0 for both parameters in the grid search optimization.

Supplementary Table 1 (CONT). Group-averaged excitation-inhibition ratio for all brain regions aggregated for NC, APOE, NC_males, NC_females, APOE_males, APOE_females, and the delta between groups

Region	*NC*	*APOE*	*Delta*	*NC_males*	*APOE_males*	*Delta*	*NC_females*	*APOE_females*	*Delta*
Left Amygdala	1.15	1.14	-1%	1.22	1.14	-7%	1.09	1.04	-5%
Left Bankssts	1.05	1.08	3%	1.04	1.08	4%	1.06	1.2	12%
Left Caudal Anterior Cingulate	0.94	0.86	-9%	1.01	0.86	-17%	0.9	0.8	-13%
Left Caudal Middle Frontal	0.97	1.07	9%	0.96	1.07	10%	0.98	1.16	16%
Left Caudate	0.97	1.02	5%	1.11	1.02	-9%	0.87	1.04	16%
Left Cuneus	1.13	1.26	10%	1.19	1.26	6%	1.08	1.1	2%
Left Entorhinal	0.95	0.94	-1%	1	0.94	-6%	0.92	0.94	2%
Left Frontal Pole	1	1.17	15%	0.98	1.17	16%	1	1.13	12%
Left Fusiform	1.04	1.15	10%	1.12	1.15	3%	0.97	1.07	9%
Left Hippocampus	0.95	1.07	11%	0.98	1.07	8%	0.9	0.98	8%
Left Inferior Parietal	1.13	1.15	2%	1	1.15	13%	1.21	1.22	1%
Left Inferior Temporal	1.01	1.02	1%	0.98	1.02	4%	1.03	1.04	1%
Left Insula	0.86	0.86	0%	0.89	0.86	-3%	0.85	0.79	-8%
Left Ithmus of the Cingulate	1.05	1.01	-4%	1.04	1.01	-3%	1.07	1.12	4%
Left Lateral Occipital	0.85	0.89	4%	0.83	0.89	7%	0.87	0.84	-4%
Left Lateral Orbitofrontal	1.17	1.28	9%	1.22	1.28	5%	1.15	1.33	14%
Left Lingual	0.95	0.91	-4%	0.96	0.91	-5%	0.95	1.07	11%
Left Medial Orbitofrontal	1.06	1.1	4%	1.07	1.1	3%	1.05	1.2	13%
Left Middle Temporal	1.22	1.14	-7%	1.13	1.14	1%	1.29	1.31	2%
Left Pallidum	1.11	1.12	1%	1.15	1.12	-3%	1.1	1.06	-4%
Left Paracentral	0.9	0.94	4%	0.93	0.94	1%	0.86	1.01	15%
Left Parahippocampus	0.91	0.97	6%	0.94	0.97	3%	0.9	0.97	7%
Left Pars Triangularis	1.03	1.18	13%	1.09	1.18	8%	0.99	1.16	15%
Left Parsopercularis	1.1	1.16	5%	1.07	1.16	8%	1.12	1.16	3%
Left Parsorbitalis	1.11	1.05	-6%	1.08	1.05	-3%	1.14	1.11	-3%
Left Pericalcarine	1.01	1.04	3%	1.05	1.04	-1%	0.98	1.05	7%
Left Postcentral	1	0.94	-6%	1.01	0.94	-7%	0.98	1.02	4%
Left Posterior Cingulate	1.15	1.27	9%	1.16	1.27	9%	1.13	1.3	13%
Left Precentral	0.79	0.86	8%	0.87	0.86	-1%	0.73	0.75	3%
Left Precuneus	1.01	1.02	1%	1.02	1.02	0%	0.99	1.14	13%
Left Putamen	1.13	1.24	9%	1.21	1.24	2%	1.08	1.14	5%
Left Rostral Anterior Cingulate	0.8	0.9	11%	0.85	0.9	6%	0.76	0.85	11%
Left Rostral Middle Frontal	0.99	1.05	6%	0.97	1.05	8%	1.01	1.09	7%
Left Superior Frontal	1.2	1.28	6%	1.09	1.28	15%	1.28	1.26	-2%
Left Superior Parietal	0.87	0.88	1%	0.91	0.88	-3%	0.83	0.97	14%
Left Superior Temporal	1.17	1.29	9%	1.12	1.29	13%	1.2	1.15	-4%
Left Supramarginal	1.1	1.19	8%	1.11	1.19	7%	1.1	1.23	11%
Left Temporal Pole	0.83	0.91	9%	0.87	0.91	4%	0.81	0.83	2%
Left Thalamus	0.81	0.79	-3%	0.86	0.8	-8%	0.76	0.76	0%
Left Transverse Temporal	0.98	0.94	-4%	0.99	0.94	-5%	0.97	1.15	16%

Supplementary Table 1 (CONT).

Region	***NC***	***APOE***	***Delta***	***NC_males***	***APOE_males***	***Delta***	***NC_females***	***APOE_females***	***Delta***
Right Ithmus of the Cingulate	1.05	1.07	2%	1	1.07	7%	1.09	1.11	2%
Right Amygdala	1.18	1.32	11%	1.2	1.32	9%	1.15	1.22	6%
Right Bankssts	1.16	1.17	1%	1.1	1.17	6%	1.2	1.37	12%
Right Caudal Anterior Cingulate	0.96	1.02	6%	0.95	1.02	7%	0.96	1.11	14%
Right Caudal Middle Frontal	0.99	1	1%	1.02	1	-2%	0.96	1	4%
Right Caudate	0.88	0.89	1%	0.94	0.89	-6%	0.84	0.86	2%
Right Cuneus	1.17	1.14	-3%	1.26	1.14	-11%	1.08	1.11	3%
Right Entorhinal	1.02	1.01	-1%	1.03	1.01	-2%	1.02	1.09	6%
Right Frontal Pole	1.04	1.16	10%	1.04	1.16	10%	1.05	1.11	5%
Right Fusiform	1.08	1.13	4%	1.13	1.13	0%	1.06	1	-6%
Right Hippocampus	1.2	1.22	2%	1.22	1.22	0%	1.16	1.2	3%
Right Inferior Parietal	1.13	1.14	1%	1.12	1.14	2%	1.14	1.3	12%
Right Inferior Temporal	1.12	1.05	-7%	1.14	1.05	-9%	1.11	1.05	-6%
Right Insula	1.01	1.04	3%	1.06	1.04	-2%	0.98	1.06	8%
Right Later Occipital	0.9	0.91	1%	0.92	0.91	-1%	0.9	0.81	-11%
Right Lateral Orbitofrontal	1.21	1.18	-3%	1.24	1.18	-5%	1.18	1.11	-6%
Right Lingual	0.99	1.07	7%	0.93	1.07	13%	1.02	1.04	2%
Right Medial Orbitofrontal	1	1.11	10%	1.05	1.11	5%	0.96	1.1	13%
Right Middle Temporal	1.23	1.31	6%	1.18	1.31	10%	1.26	1.53	18%
Right Pallidum	1.28	1.26	-2%	1.26	1.26	0%	1.3	1.16	-12%
Right Paracentral	1.06	1.14	7%	1.09	1.14	4%	1.03	1.06	3%
Right Parahippocampus	0.88	0.87	-1%	0.89	0.87	-2%	0.86	0.9	4%
Right Paricalcarine	1.09	1.15	5%	1.15	1.15	0%	1.04	1.12	7%
Right Pars Opercularis	1.19	1.29	8%	1.16	1.29	10%	1.2	1.19	-1%
Right Pars Orbitalis	1.14	1.15	1%	1.11	1.15	3%	1.15	1.14	-1%
Right Pars Triangularis	1.11	1.15	3%	1.13	1.15	2%	1.11	1.19	7%
Right Postcentral	1.12	1.17	4%	1.15	1.17	2%	1.1	1.18	7%
Right Posterior Cingulate	0.96	1.01	5%	0.97	1.01	4%	0.94	1.08	13%
Right Precentral	1.11	1.16	4%	1.12	1.16	3%	1.11	1.19	7%
Right Precuneus	1.11	1.07	-4%	1.08	1.07	-1%	1.13	1.23	8%
Right Putamen	1.46	1.36	-7%	1.56	1.36	-15%	1.38	1.29	-7%
Right Rostral Anterior Cingulate	0.95	1.08	12%	0.97	1.08	10%	0.94	1.04	10%
Right Rostral Middle Frontal	0.92	1.03	11%	0.88	1.03	15%	0.94	0.91	-3%
Right Superior Frontal	1.05	1.16	9%	1.02	1.16	12%	1.07	1.3	18%
Right Superior Parietal	0.88	0.86	-2%	0.94	0.86	-9%	0.84	0.92	9%
Right Superior Temporal	1.27	1.16	-9%	1.22	1.16	-5%	1.31	1.46	10%
Right Supramarginal	1.11	1.23	10%	1.15	1.23	7%	1.07	1.2	11%
Right Temporal Pole	0.99	0.96	-3%	1.01	0.96	-5%	0.99	0.96	-3%
Right Thalamus	0.94	1.09	14%	0.92	1.09	16%	0.94	1	6%
Right Transverse Temporal	1	1.05	5%	1.01	1.05	4%	1	1.17	15%
